# Facile electrophoretic deposition of diverse functional materials for scalable electrode and photoelectrode fabrication

**DOI:** 10.1039/d6ra05969d

**Published:** 2026-08-17

**Authors:** Madasamy Thangamuthu, Tara M. LeMercier, Emerson C. Kohlrausch, Sean Hoggett, Matthew S. Sandhu, Pekka Korhonen, Sherdil Khan, Andrea Laybourn, Anabel E. Lanterna, Jesum Alves Fernandes, Andrei N. Khlobystov

**Affiliations:** a School of Chemistry, University of Nottingham University Park Nottingham NG7 2RD UK madasamy.thangamuthu1@nottingham.ac.uk andrei.khlobystov@nottingham.ac.uk; b Institute of Physics, Universidade Federal Do Rio Grande Do Sul (UFRGS) Porto Alegre 91501-970 RS Brazil; c Institute of Process Research and Development, School of Chemistry, University of Leeds Leeds UK

## Abstract

Electrophoretic deposition (EPD) offers a scalable, solution-processable and binder-free route for assembling functional electrode films, yet its applicability across chemically diverse materials remains largely unexplored. In this study, we present a facile EPD platform for the rapid fabrication of electrodes and photoelectrodes from more than 20 functional materials, including carbon nanostructures, polymeric semiconductors, metal oxides, chalcogenides, nitrides, porous metal–organic frameworks, covalent organic frameworks and hybrid composites. Optimised deposition recipes are established for each material by controlling suspension chemistry, charging agents, applied voltage and deposition time. For photoactive materials, the photocurrent response is used as a practical descriptor to evaluate film quality and guide loading optimisation. Structural characterisation by scanning electron microscopy and transmission electron microscopy confirms uniform coatings, controlled morphologies, and intimate contact between deposited materials and conductive substrates. The versatility of the method is further validated by conformal deposition on both indium tin oxide (ITO) and porous carbon paper substrates, highlighting its compatibility with diverse electrode architectures. We also clarify the role of iodine and citric acid as charging agents for stabilising suspensions and ensuring reproducible deposition. Overall, this work demonstrates EPD as a versatile and scalable approach for integrating a wide range of functional materials into binder-free electrodes for photoelectrochemical and catalytic applications.

## Abstract 2

A broadly applicable electrophoretic deposition platform enables binder-free fabrication of diverse functional electrodes and photoelectrodes from carbon materials, semiconductors, chalcogenides, oxides, nitrides, MOFs, COFs and hybrid composites.

## Graphical abstract

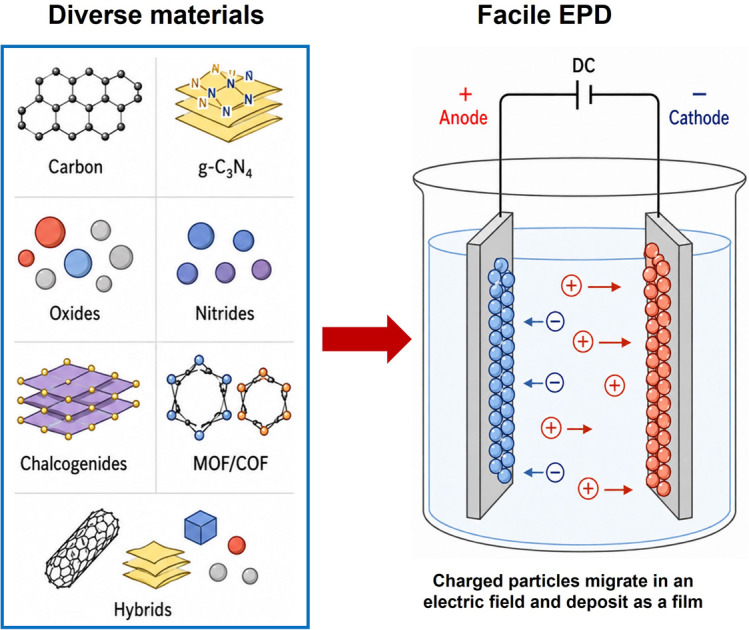

## Introduction

The development of photoelectrochemical (PEC) and electrochemical technologies relies not only on discovering active functional materials but also on integrating them into uniform, adherent, and electronically connected electrode architectures.^1–4^ Many promising materials are obtained as powders, particles, nanosheets, nanotubes, or porous frameworks, which must be converted into stable films on conductive substrates before they can be evaluated or used in practical devices. This integration challenge is particularly evident for porous framework materials, such as metal–organic frameworks (MOFs) and framework-derived composites, which are attractive for PEC applications but often suffer from low electrical conductivity, inefficient interfacial charge transport, and limited stability.^5,6^ Recent studies on BiVO_4_-based photoanodes also demonstrate that interfacial bridges, charge-transfer layers, cocatalyst integration, and directional charge management are essential for improving charge separation, surface reaction kinetics, and photoelectrode stability.^7–10^ These examples show that electrode performance depends not only on the intrinsic activity of the material but also on how effectively it is assembled into an electrically connected and accessible film. This requirement becomes even more critical when coating porous and three-dimensional substrates, such as carbon paper, metal meshes, and structured current collectors, where conformal coverage, open pathways for electrolyte access, and strong interfacial contact must be achieved simultaneously. Therefore, developing a general, binder-free, and scalable deposition strategy is key to enabling the practical implementation of diverse functional materials in electrochemical and photoelectrochemical devices.

Conventional electrode fabrication methods, including drop-casting,^11^ spin-coating,^12^ doctor-blading,^13^ hydrothermal growth,^14^ and vacuum-based deposition,^15,16^ offer important advantages but are often difficult to generalise across different classes of materials. For instance, slurry-based methods such as doctor-blading and related large-area coating approaches are widely used in commercial electrode manufacturing, but they typically require binders, conductive additives, and carefully optimised ink rheology, which can obscure active sites and introduce additional charge transport resistance. Spin-coating and drop-casting are simple laboratory methods, but they are less suitable for conformal coating of porous or three-dimensional substrates. Hydrothermal and direct-growth methods can produce strongly attached films but usually require material-specific precursor chemistry and growth conditions. Vacuum-based methods provide high-quality coatings, but often involve higher cost, limited material compatibility and more complex instrumentation. These limitations make it challenging to compare diverse materials under consistent fabrication conditions and hinder the scalable preparation of complex electrode architectures.

In contrast to the conventional methods discussed above, electrophoretic deposition (EPD) offers a complementary approach because it uses an applied electric field to drive charged particles from suspension onto a conductive substrate. This enables rapid, binder-free film formation under mild conditions, with film loading controlled by deposition voltage, time, particle concentration, and electrode spacing.^17^ Unlike many coating methods that rely primarily on ink viscosity, precursor reactivity or line-of-sight deposition, EPD can directly assemble particulate materials onto both planar and porous conductive substrates. This makes it particularly attractive for coating films on carbon paper, metal meshes and other complex electrode architectures where conformal coverage and electronic contact are essential.^18,19^ Despite these advantages, the general applicability of EPD across chemically and structurally diverse photoactive and support materials has not been systematically demonstrated.

Here, we report a facile EPD platform for fabricating electrodes and photoelectrodes from more than 20 functional materials, spanning carbon-based conductors, polymeric semiconductors, metal oxides, nitrides, chalcogenides, MOF/COF, and hybrid composites. Optimised deposition conditions are established for each material class by controlling suspension chemistry, charging agents, applied voltage, and deposition time. For photoactive materials, photocurrent measurement is used as a practical descriptor of electrode quality, providing a rapid means to assess film integrity, interfacial charge transport, and optimal material loading. Scanning electron microscopy (SEM) and transmission electron microscopy (TEM) analysis further confirm uniform coating, conformal coverage, and intimate interfacial contact between the deposited materials and substrates. The successful deposition of these diverse materials on both ITO and carbon paper demonstrates the versatility and scalability of EPD as a general route for preparing functional electrodes, photoelectrodes, and coated support materials for energy conversion and catalytic applications.

## Materials and methods

### Materials, chemicals and reagents

Carbon black (Vulcan XC 72R), sodium borohydride, CeO_2_, TiO_2_, MoS_2_, WS_2_, and Ta_2_O_5_ were purchased from Sigma-Aldrich and used as received. Graphitic nanofibers (GNFs, PyroGraf PR-24-XT-HHT) and multi-walled carbon nanotubes (MWCNTs, NanoLab PD30) were used as received. To enhance surface charge generation and improve suspension stability during EPD, both GNF and MWCNT powders were separately oxidised by refluxing in a HNO_3_/H_2_SO_4_ mixture (1 : 3 v/v) at 130 °C for 30 min. Following oxidation, the suspensions were diluted with 250 mL of deionised water and filtered using a PTFE membrane filter. The collected solids were subsequently washed with deionised water (250 mL) and acetone (250 mL) to remove residual acids and impurities. The purified oxidised materials were dried under vacuum in a desiccator for 24 h and then ground using a mortar and pestle to obtain fine powders. Acetone, ethanol, isopropanol and other chemicals used in this study were purchased from commercial sources and used without further purification.

### Synthesis of carbon nitrides

Graphitic carbon nitride (g-C_3_N_4_) was synthesised using the thermal polycondensation method.^20^ In brief, 8 g of melamine was heated in a quartz crucible at 350 °C for 30 minutes in air, at a heating rate of 5 °C min^−1^, followed by heating at 550 °C for 3 hours in air. The resulting yellow powder was then cooled to room temperature inside the furnace and ground to obtain bulk-C_3_N_4_. Subsequently, g-C_3_N_4_ was obtained by annealing the bulk-C_3_N_4_ at 500 °C for 2 hours in air with a heating rate of 5 °C min^−1^.

B-doped carbon nitride was synthesised by dissolving 0.1 g of BH_3_NH_3_ in 10 mL of water under stirring for 5 minutes. Then, 3 g of dicyandiamide was added to it, and the mixture was heated in an oil bath at 80 °C until the water was evaporated and a white solid formed. The obtained solid was placed in a quartz crucible covered with perforated aluminium foil and heated to 600 °C at a heating rate of 2.5 °C min^−1^, followed by holding at this temperature for 4 hours under Ar flow (200 mL min^−1^). The sample was then allowed to cool naturally to room temperature inside the furnace, yielding a compressed dark orange powder, which was ground to a fine powder using a mortar and pestle.

S-doped carbon nitride was synthesised by heating 5 g of thiourea in a quartz crucible with a loosely fitted lid to 550 °C (3 °C min^−1^) for 5 h. The resulting powder was then cooled to room temperature inside the furnace and ground into a fine powder to obtain S-C_3_N_4_.

### Synthesis of tungsten oxide

Tungsten oxide (WO_3_) powder was prepared *via* a room-temperature precipitation route adapted from our previous report.^21^ Briefly, 0.8 g of sodium tungstate dihydrate (Na_2_WO_4_·2H_2_O) was dissolved in 20 mL of deionised water under stirring. Concentrated hydrochloric acid (2 mL, 12 M) was then introduced dropwise while maintaining continuous stirring, resulting in the gradual formation of a pale-yellow suspension. The reaction mixture was stirred for a further 1 h to complete precipitation. The resulting solid was isolated by centrifugation (8000 rpm, 5 min) and repeatedly washed with water and ethanol until the supernatant reached approximately neutral pH. The obtained precipitate was dried at 70 °C overnight and ground into a fine powder using a mortar and pestle. Finally, the powder was thermally treated in air at 400 °C for 2 h (heating rate: 10 °C min^−1^) to obtain monoclinic WO_3_.

### Synthesis of tantalum nitride

Tantalum nitride (Ta_3_N_5_) was synthesised by nitridation of commercial Ta_2_O_5_ nanoparticles using a previously reported method.^22^ Briefly, Ta_2_O_5_ powder (200 mg) was placed in an alumina boat and inserted into a horizontal quartz tube furnace. Ammonolysis was then carried out at 900 °C for 3 h under a continuous NH_3_ flow of 125 sccm, with heating and cooling rates of 5 °C min^−1^. After the reaction, the samples were cooled under constant NH_3_ flow, washed with deionised water, and dried before further use.

### Synthesis of NU-1000 MOF

NU-1000 MOF was synthesised using a microwave-assisted method.^23^ Briefly, ZrOCl_2_ (200 mg) and benzoic acid (3.0 g) were dissolved in DMF (40 mL) in a 100 mL microwave reaction vessel. The mixture was heated to 100 °C for 30 min using a multimode microwave reactor (maximum power: 900 W) to generate the Zr-node precursor solution. After cooling to room temperature, TBAPy (80 mg) and trifluoroacetic acid (500 µL) were added to the reaction mixture. The resulting solution was then heated to 150 °C at a ramp rate of 25 °C min^−1^ and held for 60 min. After cooling, the solid product was collected by centrifugation (4000 rpm, 30 min, 5 °C) and washed twice with DMF. To remove residual benzoate modulators, the obtained solid was dispersed in DMF (40 mL) containing concentrated HCl (2 mL), followed by sonication for 15 min and soaking for 1 h before centrifugation. The purified NU-1000 was subsequently washed three times with acetone for solvent exchange and dried in air at 80 °C overnight to obtain the final product.

### Synthesis of COF, COF/MWCNTs, and COF/GNFs

COF-366-Co was synthesised using a modified literature method.^24^ Briefly, 5,10,15,20-tetra(4-aminophenyl)porphyrin cobalt(ii) (Co-TAPP) (18 mg, 0.025 mmol), benzene-1,4-dicarboxylaldehyde (10 mg, 0.075 mmol), mesitylene (1 mL), ethanol (1 mL), dioxane (2 mL) and acetic acid (0.25 mL, 6 M) were added to a Schlenk tube. The mixture was sonicated for 1 h, flash-frozen in liquid nitrogen, and subjected to three freeze–pump–thaw cycles before being sealed under argon. The reaction mixture was heated to 120 °C for 72 h, resulting in the formation of a dark purple precipitate. The product was filtered using a PTFE membrane filter and washed with methanol, water and chloroform to obtain Co-TAPP–BDA-COF (COF-366-Co, 18.4 mg, 84% yield).

COF-366-Co/MWCNT was synthesised using a similar procedure.^25^ 5,10,15,20-tetra(4-aminophenyl)porphyrin cobalt(ii) (Co-TAPP) (5 mg, 0.007 mmol), benzene-1,4-dicarboxylaldehyde (5 mg, 0.038 mmol), MWCNTs (3.34 mg), mesitylene (0.28 mL), ethanol (0.28 mL), dioxane (0.56 mL) and acetic acid (0.07 mL, 6 M) were added to a Schlenk tube. The mixture was sonicated for 1 h, flash-frozen in liquid nitrogen, subjected to three freeze–pump–thaw cycles and sealed under argon. The mixture was then heated at 120 °C for 72 h, resulting in the formation of a dark purple precipitate, which was filtered using a PTFE membrane filter and washed with methanol, water and chloroform. This yielded COF-366-Co/MWCNTs (2.46 mg, 25% yield).

The COF-366-Co/GNF was synthesised using the same procedure with adjusted quantities of benzene-1,4-dicarboxylaldehyde (2 mg, 0.015 mmol) and GNFs (1 mg), while all other steps were conducted as described for COF-366-Co/MWCNT. The final COF-366-Co/GNFs product was obtained as a dark-purple solid (2.3 mg, 38% yield).

### Metal loading by controlled magnetron sputtering

The Cu and Pt metal atoms were deposited on powder samples of selected functional materials using an AJA magnetron sputtering system equipped with a custom-built stirring sample holder. Sputtering was carried out at a working pressure of 3 mTorr under argon gas. A voltage of 370 V and a current of 16 mA were applied to generate the metal atom flux for the desired deposition time. The stirring holder ensured continuous movement of the powder during sputtering to promote more homogeneous metal deposition.

### Electrophoretic deposition of films

EPD was carried out using a two-electrode configuration connected to a DC power supply. Two conductive substrates, ITO or carbon paper, were placed parallel to each other at a fixed distance of 2 cm and immersed in the prepared suspension. The target material was dispersed in the selected solvent with or without a charging agent, followed by ultrasonication to obtain a stable suspension. Before EPD coating, ITO substrates were cleaned by sequential ultrasonication in deionised water and IPA for 15 min each, followed by UV-ozone treatment for 30 min. Carbon paper substrates were washed with acetone and deionised water and then annealed at 300 °C for 2 h in air to remove organic residue from the manufacturing process. EPD coating was performed under a constant applied voltage for a defined time, depending on the material-specific optimisation conditions summarised in Table S1. The deposition electrode polarity depended on the surface charge of each material, with positively charged particles depositing on the cathode and negatively charged particles depositing on the anode. After deposition, the coated electrodes were removed from the suspension and gently rinsed with the corresponding solvent to remove loosely attached particles. Unless otherwise stated, inorganic semiconductors and carbon-based films were thermally treated at 300 °C for 2 h in air, whereas MOF/COF-based films were dried at 60 °C overnight before further characterisation. Film loading was estimated from the mass difference of the substrate before and after deposition and normalised to the geometric electrode area.

### Preparation of films by drop-casting and spin-coating

For comparison with EPD, g-C_3_N_4_ films were prepared on ITO by drop-casting and spin-coating. For drop-casting, 20 µL of g-C_3_N_4_ ink was deposited onto the ITO substrate and dried; this process was repeated three times to improve surface coverage. For spin-coating, the g-C_3_N_4_ ink was deposited onto ITO and spun under the selected conditions. The resulting films were dried before photoelectrochemical testing.

### Tape-peel adhesion test

Tape-peel adhesion tests were carried out on representative EPD-coated electrodes. Adhesive tape was placed on the coated area, gently pressed for 2 min, and then peeled off under the same conditions. Optical photographs were recorded before and after tape removal. The electrode mass was measured before and after the tape-peel test using a microbalance. Film retention was calculated from the mass difference before and after tape peeling.

### Characterisation

Scanning electron microscopy (SEM) images were obtained using a JEOL 7000F Field Emission Gun microscope operated at 10 kV e-beam. The high-resolution transmission electron microscopy (HR-TEM) images were obtained using a JEOL FEG-TEM microscope equipped with a single-electron detector Gatan K3-IS camera operated at 200 kV. TEM samples were prepared by dispersing the samples in propan-2-ol using ultrasonication, followed by drop-casting the suspension onto Au grids mounted on “holey” carbon films (Agar). All TEM images were processed using Gatan Digital Micrograph.

Ultraviolet-visible (UV-vis) diffuse reflectance spectra were recorded using an Agilent Cary 5000 UV-vis NIR absorption spectrometer equipped with a DRA-900 InGaAs integrating sphere, and optical band gaps were estimated from Tauc plots. Mott–Schottky measurements were performed in 0.5 M Na_2_SO_4_ electrolyte at 1000 Hz with an AC amplitude of 10 mV, and the flat-band potential was obtained by extrapolating the linear region of the 1/*C*^2^*versus* potential plot.

### Photoelectrochemical characterisation

Photocurrent measurements were performed in a three-electrode configuration using the EPD-coated electrode as the working electrode, Ag/AgCl as the reference electrode, and graphite as the counter electrode. Measurements were carried out using an IVIUM electrochemical potentiostat. Unless otherwise stated, photoanode measurements were conducted in 0.5 M KHCO_3_ containing 10 vol% triethanolamine (TEOA) as a hole scavenger. WO_3_ electrodes were tested in 0.5 M KHCO_3_ containing 10 vol% methanol, while Ta_3_N_5_ electrodes were measured in 0.1 M KOH. For B-doped C_3_N_4_ photocathode measurements, the electrolyte was saturated with CO_2_ before measurement. The observed potentials against the Ag/AgCl reference were converted to the RHE scale using the Nernst equation: *E*_(RHE)_ = *E*_(Ag/AgCl)_ + 0.197 + 0.0596 × pH. The photoelectrodes were illuminated using an LED solar simulator (Ossila) under standard one sun conditions (100 mW cm^−2^).

## Results and discussion

### Design and principle of the electrophoretic deposition

**Fig. 1:**
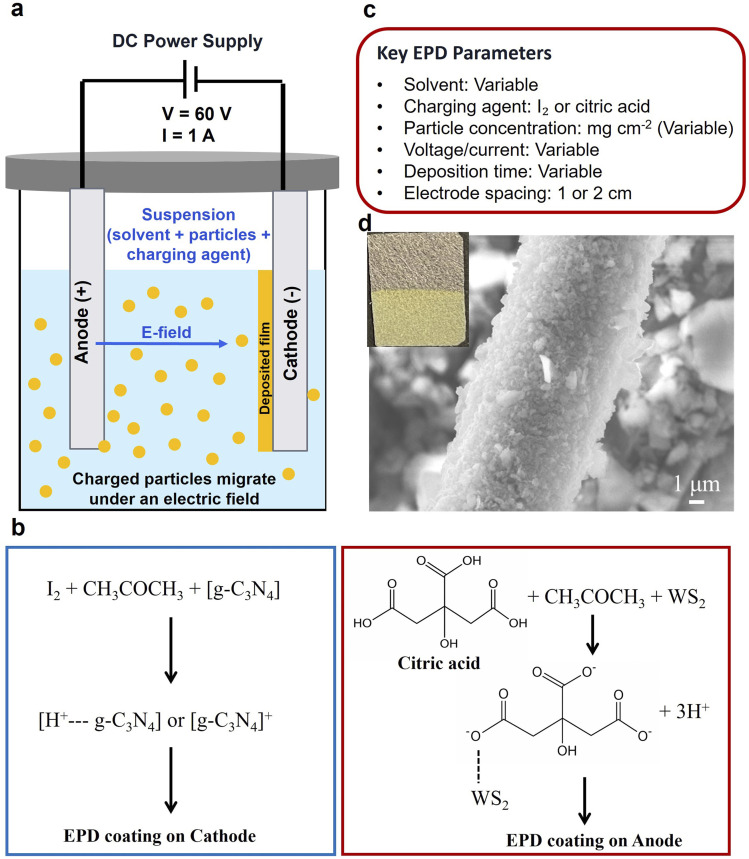
Design principle and key parameters of electrophoretic deposition. (a) Schematic illustration of EPD, showing the migration of charged particles under an applied electric field and deposition onto the oppositely biased conductive substrate, (b) proposed charging mechanisms for acetone + I_2_ and acetone + citric acid-based EPD suspensions. These mechanisms are consistent with the deposition polarity observed in this work, (c) key parameters controlling film formation, including solvent, charging agent, particle concentration, applied voltage/current, deposition time, and electrode spacing, (d) representative g-C_3_N_4_ film deposited on porous carbon paper, demonstrating conformal coating of a three-dimensional conductive substrate.

EPD is a solution-based technique in which charged particles dispersed in a liquid medium migrate under an applied electric field and deposit onto an oppositely charged conductive substrate. In this work, we establish a general EPD platform for fabricating uniform, binder-free films across a wide range of material classes. As illustrated in Fig. 1a, a stable suspension of the target material is subjected to a constant electric field between two electrodes, driving the directional particle migration and film formation on the oppositely charged electrode. Achieving stable suspensions with sufficient surface charge is critical for reproducible deposition. To this end, charging agents such as iodine (I_2_) and citric acid were introduced to generate surface charge and enhance dispersion stability across chemically diverse systems. In acetone-based suspensions containing I_2_, previous EPD studies have demonstrated proton generation *via* acetone keto–enol/iodination reactions (eqn (1) and (2)), followed by proton adsorption on particle surfaces.^20,26^

1 CH_3_COCH_3_ ⇌ CH_3_C(OH)CH_2_

2 CH_3_C(OH)CH_2_ + I_2_ → CH_3_COCH_2_I + H^+^ + I^−^

This mechanism promotes migration of positively charged particles towards the cathode. In contrast, citric acid can act as a carboxylate-based charging and stabilising agent in acetone-based EPD suspensions. Citrate/carboxylate species adsorb on particle surfaces, imparting negative surface charge and improving colloidal stability, which favours anodic deposition.^27^ These mechanisms are consistent with the cathodic deposition observed for I_2_/acetone-based suspensions and the anodic deposition observed for citric acid-stabilised MoS_2_ and WS_2_ suspensions in this work (Fig. 1b).

To further evaluate the role of charging-agent concentration, representative experiments were carried out for I_2_ and citric-acid-assisted EPD systems. For g-C_3_N_4_ suspensions in acetone + I_2_, a low I_2_ concentration of 5 mg in 30 mL acetone (0.65 mM) resulted in weak deposition, indicating insufficient surface charging. Increasing the I_2_ concentration to 10 mg in 30 mL acetone (1.31 mM) produced the most uniform and reproducible coating. However, excessive I_2_ concentration of 50 mg in 30 mL acetone (6.5 mM) reduced suspension stability and led to less uniform film formation. Similarly, for citric-acid-stabilised WS_2_ suspensions, among the various concentrations tested, 2.5 mM citric acid provided the best EPD-coating. These results show that charging agent concentration is also a critical parameter in EPD and that the optimum dosage depends on material-specific surface chemistry, suspension stability, and electrophoretic mobility.

The EPD process allows direct assembly of particulate materials into adherent films without the use of polymeric binders, thereby preserving surface accessibility and electronic connectivity. Importantly, film thickness and material loading (mg cm^−2^) can also be systematically tuned by adjusting key deposition parameters, including particle concentration, applied voltage or current, deposition time, and electrode spacing (Fig. 1c).

The versatility of the platform is further demonstrated by its compatibility with both planar and porous conductive substrates, enabling conformal coating on electrode architectures relevant to practical devices. As a representative example, g-C_3_N_4_ was deposited as a uniform, binder-free film on carbon paper, exhibiting controlled film growth and a tuneable photocurrent response with deposition conditions (Fig. 1d). The following sections demonstrate how this strategy can be adapted to different material classes by tuning suspension chemistry, charging agent, voltage, deposition time, and substrate configuration. The detailed optimised EPD conditions for each material are summarised in Table S1; therefore, the following sections focus on the key deposition behaviour, morphology, substrate compatibility, and functional relevance of each material class rather than repeating the full optimisation process. Photocurrent measurements are used as a practical indicator of film quality, loading optimisation, and functional photoelectrode behaviour rather than as a direct benchmark against literature photoelectrodes, since photocurrent values depend strongly on material composition, film thickness, substrate, electrolyte, sacrificial reagent, illumination conditions, and applied potential.

### Carbon-based conductors

**Fig. 2:**
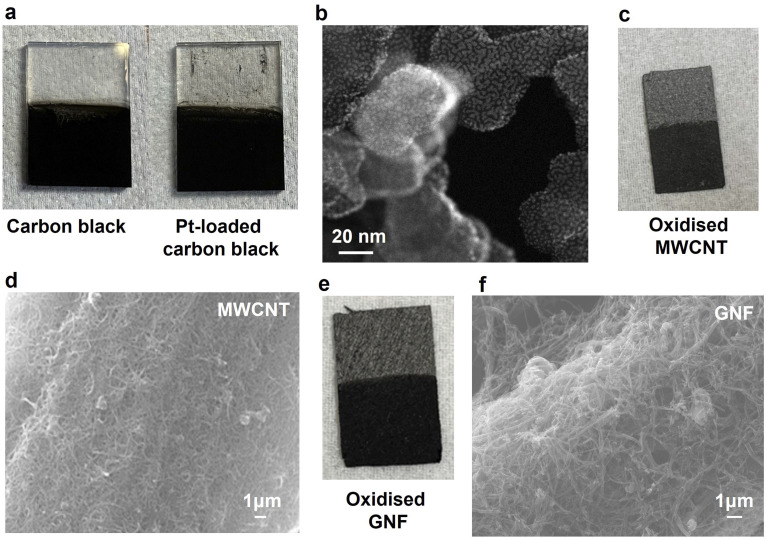
EPD of carbon-based conductive materials. (a) Photograph of carbon black and Pt/carbon black deposited on ITO, (b) HR-TEM image of the EPD-coated Pt/carbon black film, (c) photograph and (d) SEM image of oxidised MWCNTs deposited on carbon paper, (e) photograph and (f) SEM image of oxidised GNFs deposited on carbon paper. The images show that surface functionalisation and solvent selection are important for obtaining uniform carbon coatings.

Carbon-based materials, including carbon black and Pt-loaded carbon black, were first examined to assess the applicability of EPD to conductive particulate systems. Both materials were deposited on ITO from acetone-based suspensions to evaluate the role of I_2_ and water in charge generation. In the absence of I_2_, no measurable deposition was observed over a wide range of applied voltages (20–60 V) and deposition times (2–15 min) (Fig. S1a), indicating insufficient surface charge and particle mobility. The introduction of I_2_ enabled partial deposition; however, the resulting films were sparse and non-uniform (Fig. S1b). Uniform and adherent carbon black and Pt-loaded carbon black films were obtained only after adding 5 vol% water to the acetone + I_2_ suspension, under 60 V for 5 min (Fig. 2a, S1c and d). This improvement is attributed to enhanced ion dissociation, surface protonation, and suspension stability, consistent with the I_2_/acetone-assisted positive charging mechanism discussed in the previous section.^20^ The added water increases medium polarity and stabilises ionic species, thereby improving particle mobility and film uniformity. HR-TEM analysis further confirms the homogeneous distribution of Pt-loaded carbon black within the deposited film (Fig. 2b).

As a second representative example, MWCNTs were deposited onto carbon paper electrodes. Initial screening showed that non-oxidised MWCNTs produced either negligible or non-uniform deposition from ethanol, acetone, and ethanol + acetone mixture, both in the presence and absence of I_2_ (Fig. S2a–c). To enhance surface charging, MWCNTs were oxidised by refluxing in a HNO_3_/H_2_SO_4_ acid mixture, introducing oxygen-containing functional groups such as –COOH and –OH onto the nanotube surface. After oxidation, MWCNTs dispersed in an acetone + ethanol (1 : 1) mixture with I_2_ were deposited uniformly on carbon paper at 20 V for 1 min (Fig. 2c and S2d). This improvement is attributed to the increased density of surface functional groups, which promote effective proton adsorption and improve surface charge generation in the presence of I_2_, thereby increasing electrophoretic mobility and enabling uniform film formation under milder deposition conditions. The SEM image confirms homogeneous MWCNT coverage across the carbon fibres of the carbon paper substrate (Fig. 2d).

A similar requirement for surface functionalisation was observed for graphitic carbon nanofibers (GNFs). Non-oxidised GNFs showed negligible deposition under the investigated conditions (Fig. S3a), indicating insufficient surface charge for electrophoretic transport. After oxidation, GNFs suspensions prepared in acetone with I_2_ exhibited uniform and adherent film formation on carbon paper at 60 V for 1 min (Fig. 2e and S3b). Unlike MWCNTs, the acetone + ethanol mixed solvent did not improve GNF deposition quality (Fig. S3c), highlighting morphology-dependent differences in suspension behaviour. These results indicate that, even within the same broad material class, morphology and surface chemistry strongly influence suspension stability and charge development during EPD. The SEM analysis confirms homogeneous GNFs coverage on carbon paper (Fig. 2f). The electrochemical impedance spectroscopy (EIS) measurements were further employed to evaluate the interfacial charge-transfer behaviour of the EPD-coated MWCNT/CP and GNF/CP electrodes. The MWCNT/CP electrode exhibits a charge-transfer resistance of 700 Ω, while the GNF/CP electrode shows a slightly lower of 670 Ω (Fig. S4a). Both values are substantially lower than that of pristine carbon paper (2200 Ω), indicating that the carbon-based coatings significantly enhance electron transport at the electrode–electrolyte interface. These results confirm that both MWCNT and GNF films formed by EPD significantly enhance electrical communication and charge-transfer kinetics between the electrode and electrolyte.

These results show that EPD is a versatile method for fabricating conductive carbon coatings on both planar ITO and porous carbon paper substrates. In particular, MWCNT and GNF coatings on carbon paper form interconnected conductive scaffolds that enhance electrical connectivity, increase surface area, and provide robust supports for integrating semiconductors, molecular catalysts, and metal-based active sites.

### Polymeric semiconductors (g-C_3_N_4_ family)

**Fig. 3:**
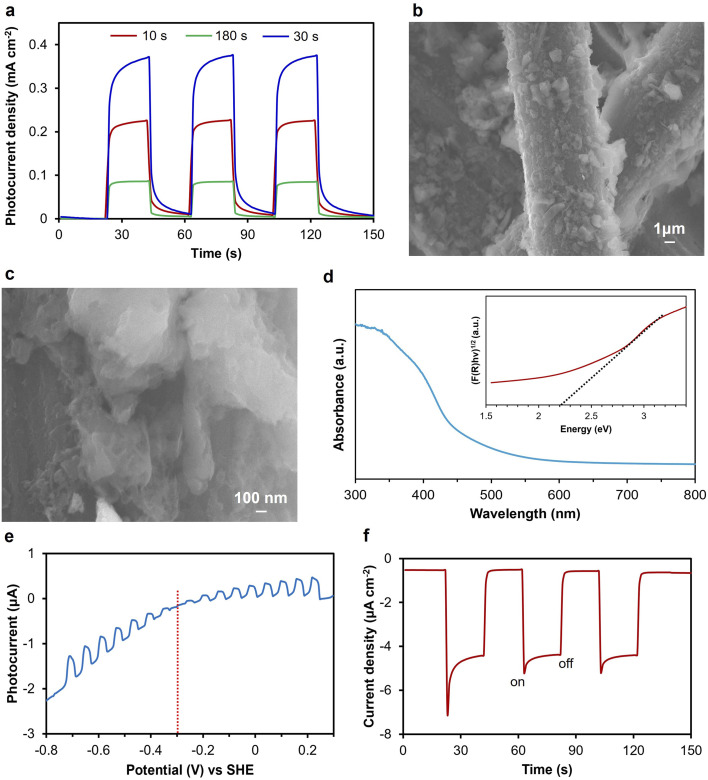
EPD-fabricated carbon nitride photoelectrodes. (a) Photocurrent responses of g-C_3_N_4_ films deposited on carbon paper for 10, 30, and 180 s, measured in 0.5 M KHCO_3_ containing 10% triethanolamine under simulated solar irradiation, (b and c) low- and high-magnification SEM images of EPD-coated B-doped C_3_N_4_ on carbon paper, (d) UV-vis absorbance spectra of the B-doped C_3_N_4_, with the corresponding Tauc plot shown in the inset, (e) the photocurrent onset potential measurement of B-doped C_3_N_4_, (f) cathodic photocurrent response of B-doped C_3_N_4_ measured in 0.5 M KHCO_3_ saturated with CO_2_.

The applicability of the EPD platform was further examined for polymeric semiconductors, including g-C_3_N_4_, and doped variants such as B- and S-doped C_3_N_4_. Suspensions were prepared in water, isopropanol (IPA), and acetone, both with and without I_2_. Among the tested conditions, acetone + I_2_ consistently produced the most uniform and adherent film on carbon paper, although the optimum suspension concentration, deposition voltage and deposition time varied depending on the material (Fig. S5a). For pristine g-C_3_N_4_, a uniform thin film was obtained from a 2 mg mL^−1^ suspension at 60 V for 30 s, which gave the highest photocurrent response (Fig. 3a). Lower deposition times resulted in insufficient film coverage and reduced light absorption, while longer deposition times produced thicker films that increased charge-transport resistance and recombination losses, as evidenced by the reduced photocurrents. This optimised EPD method was also extended to Cu-loaded g-C_3_N_4_, yielding a uniform film on carbon paper at 50 V for 90 s, suggesting that metal-modified polymeric semiconductors can also be processed using the EPD approach. The resulting g-C_3_N_4_/CP and Cu/g-C_3_N_4_/CP electrodes can be used as photoanodes for water oxidation and alcohol or organic oxidation reactions, offering potential routes to value-added chemical production. A direct comparison with drop-casting and spin-coating is discussed later in the practical applicability section using g-C_3_N_4_/ITO as a representative photoactive material.

The same deposition strategy was successfully extended to doped carbon nitride systems. A homogeneous S-doped C_3_N_4_ film was obtained from a 3 mg mL^−1^ suspension at 50 V for 2 min, while an optimised B-doped C_3_N_4_ film on carbon paper was prepared from a 5 mg mL^−1^ suspension at 60 V for 2 min deposition (Fig. S5b and c). These results indicate that chemical modification of the carbon nitride framework does not hinder EPD processability, although material-specific optimisation of concentration, voltage and deposition time remains necessary.

Since the morphology and photoelectrochemical properties of g-C_3_N_4_ and S-doped C_3_N_4_ have been discussed in previous studies,^20,28,29^ the present work focuses on B-doped C_3_N_4_ as a representative doped polymeric semiconductor. Low-magnification SEM imaging of EPD-coated B-doped C_3_N_4_ on carbon paper reveals conformal coverage over the carbon fibres (Fig. 3b), indicating that the EPD process enables uniform coating over the porous electrode architecture. At higher magnification, the film consists of aggregated nanoscale fragments forming a rough and porous surface (Fig. 3c), which is expected to increase the effective surface area and facilitate charge transfer at the electrode–electrolyte interface. The close contact between the deposited layer and the carbon fibres also supports effective electrical connectivity.

The crystallinity of B-doped C_3_N_4_ was evaluated by X-ray diffraction (XRD) and compared with pristine g-C_3_N_4_ (Fig. S6). Pristine g-C_3_N_4_ shows characteristic diffraction peaks at 2*θ* = 13.1° and 27.5°, corresponding to the in-plane (100) structural motif of tri-*s*-triazine units and the interlayer (002) stacking reflection, respectively. After B-doping, the (002) peak shifts slightly to a lower angle, indicating an increase in interlayer spacing from 0.324 to 0.330 nm. This expansion is attributed to boron incorporation into the carbon nitride framework, which weakens interlayer interactions and introduces structural distortion. In addition, the reduced intensity of the (100) peak suggests partial disruption of in-plane tri-*s*-triazine ordering. These XRD changes confirm the successful formation of B-doped C_3_N_4_. The UV-vis absorbance spectrum of the B-doped C_3_N_4_ thin film exhibits strong absorption in the visible region (Fig. 3d), while the corresponding Tauc plot gives an optical bandgap of 2.2 eV (inset of Fig. 3d), significantly narrower than that of pristine g-C_3_N_4_ (2.7 eV).^29^ Mott–Schottky analysis exhibits a negative slope in the linear region, confirming the p-type semiconducting nature of the B-doped C_3_N_4_ (Fig. S7). From the flat-band potential of approximately +1.9 V *vs.* SHE and the optical bandgap of 2.2 eV (Fig. S8), the conduction band position was estimated to be −0.3 V *vs.* SHE, consistent with the photocurrent onset potential measurement (Fig. 3e). When tested in CO_2_-saturated 0.5 M KHCO_3_ electrolyte, the B-doped C_3_N_4_ film on carbon paper electrode exhibited a pronounced cathodic photocurrent response under illumination (Fig. 3f), confirming efficient light-driven electron transfer under CO_2_ reduction conditions. Together with its suitable negative conduction band position, this response indicates that the EPD-fabricated B-doped C_3_N_4_ film is a promising photocathode platform for photoelectrochemical CO_2_ reduction.

### Metal oxides and nitrides

The versatility of the EPD method was further demonstrated for inorganic semiconductors, including TiO_2_ (anatase and P25), WO_3_, CeO_2_, and Ta_3_N_5_. Despite differences in particle size, density, and surface chemistry, these materials were successfully processed into uniform, adherent films using acetone + I_2_-based suspensions at varied deposition voltages and times.

**Fig. 4:**
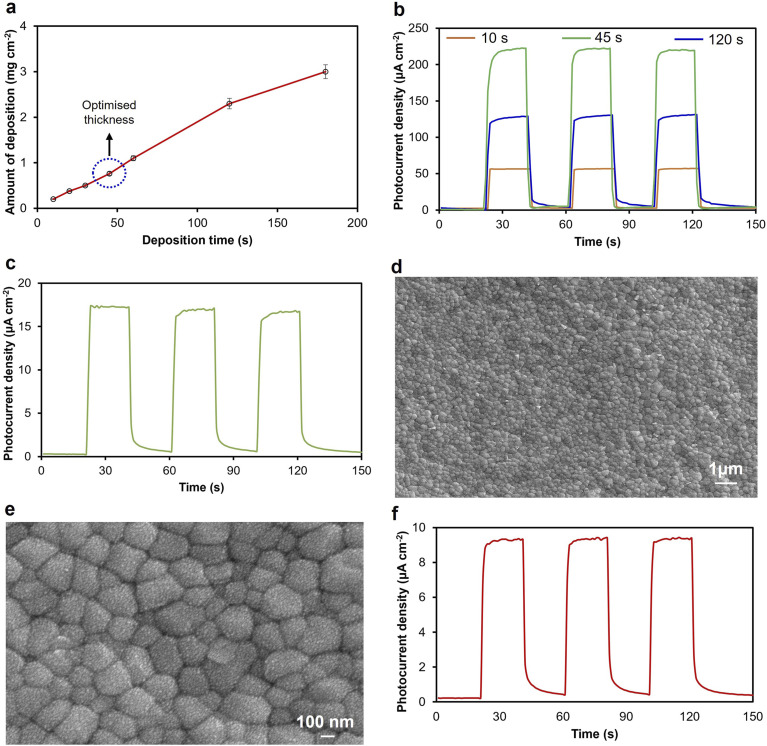
EPD of oxide and nitride semiconductor photoelectrodes. (a) Effect of TiO_2_ deposition time on film loading, showing the optimised deposition time, (b) photocurrent responses of TiO_2_ film deposited on ITO for 10, 45, and 120 s, measured in 0.5 M KHCO_3_ containing 10% triethanolamine under simulated solar irradiation, (c) photocurrent response of the WO_3_ film deposited on carbon paper measured in 0.5 M KHCO_3_ containing 10% methanol, (d) low- and (e) high-magnification SEM images of Ta_3_N_5_ film deposited on ITO substrate, (f) photocurrent response of the Ta_3_N_5_ film on ITO measured in 0.1 M KOH.

Commercial anatase and P25 powders were deposited at relatively low voltage, with film loading controlled by deposition time (Fig. 4a). The highest photocurrent response was obtained for P25 films deposited on ITO substrate at 10 V for 45 s (Fig. 4b). Shorter deposition times, such as 10 s, likely resulted in incomplete surface coverage and limited light absorption, whereas longer deposition times, such as 120 s, produced thicker films that increased charge-transport resistance and promoted recombination losses, consistent with the trends observed for g-C_3_N_4_ systems.

For WO_3_, the synthesised material required a high deposition voltage, producing uniform coatings on carbon paper at 50 V for 60 s. The higher voltage required compared with TiO_2_ is likely associated with differences in particle density, surface charge development, and electrophoretic mobility. The UV-vis absorption spectrum of the EPD-coated WO_3_ film on ITO shows strong absorption in the UV-visible region (Fig. S9), with an absorption edge extending to approximately 450–470 nm, confirming the visible-light-responsive nature of the deposited WO_3_ photoelectrode. The resulting WO_3_-coated carbon paper electrode exhibited a stable anodic photocurrent response (Fig. 4c), confirming effective film formation and charge transport within the EPD-coated layer. Together, the TiO_2_ and WO_3_ results show that EPD-fabricated oxide films can be used as photoanodes for water oxidation and related oxidative transformations.

CeO_2_ was investigated as a representative oxide support material and successfully deposited on a porous carbon paper substrate. Homogeneous and conformal CeO_2_ coatings were obtained at 50 V for 30 s (Fig. S10a), showing that EPD is not limited to photoactive semiconductors but can also be extended to functional support materials relevant for catalytic and hybrid electrode architectures.

The method was further extended to Ta_3_N_5_, a visible-light-active nitride semiconductor. Ta_3_N_5_ was successfully deposited on both ITO and carbon paper substrates at 40 V for 2 min (Fig. S10b and c). SEM analysis of the Ta_3_N_5_-coated ITO electrode reveals a uniform and continuous film. The low-magnification SEM image confirms homogeneous coverage across the substrate without significant cracking, peeling, or exposed ITO regions (Fig. 4d), while the high-magnification image shows densely packed granular Ta_3_N_5_ particles with good interparticle connectivity (Fig. 4e). This compact yet textured morphology, together with anodic photocurrent response, confirms effective film formation and photoelectrochemical activity (Fig. 4f).

EIS was further used to assess interfacial charge-transfer behaviour in representative EPD-coated photoelectrodes. The Nyquist plots of g-C_3_N_4_/CP, WO_3_/CP and WO_3_/g-C_3_N_4_/CP show that the WO_3_/g-C_3_N_4_ heterojunction electrode exhibits a smaller semicircle diameter than the individual g-C_3_N_4_/CP and WO_3_/CP electrodes (Fig. S4b), indicating reduced charge-transfer resistance and improved interfacial charge transport. This behaviour supports the formation of effective electrical contact between WO_3_, g-C_3_N_4_ and the conductive carbon paper substrate. Similarly, Cu/TiO_2_/CP shows a lower charge-transfer resistance than TiO_2_/CP, suggesting that Cu incorporation improves electrical conductivity and facilitates charge transfer within the EPD-coated electrode. The same trend was observed for CeO_2_-based electrodes, where Ru/CeO_2_/CP exhibited a smaller semicircle than CeO_2_/CP, confirming that Ru loading enhances interfacial charge-transfer kinetics. Overall, these EIS results support that binder-free EPD produces electrodes with effective substrate–film contact and accessible charge-transfer pathways.

Reproducibility of the EPD-fabricated photoelectrodes was evaluated using Ta_3_N_5_/CP as a representative system. Three independently prepared Ta_3_N_5_/CP electrodes fabricated under identical EPD conditions exhibited comparable photocurrent densities, with only small variation between electrodes (Fig. S11a). This confirms good electrode-to-electrode reproducibility of the EPD coating process. Repeated-measurement stability was further assessed by testing the same Ta_3_N_5_/CP electrode three times per day on day 1, day 3, day 5 and day 7 (Fig. S11b). The photocurrent response remained similar across repeated runs and over the seven day period, indicating stable photoelectrochemical behaviour and good measurement repeatability. These results support the reproducibility of the binder-free EPD process and the reliability of the resulting Ta_3_N_5_/CP photoelectrodes.

Overall, these results demonstrate that EPD is a robust and adaptable strategy for fabricating oxide- and nitride-based photoelectrodes. The successful deposition of TiO_2_, WO_3_, CeO_2_, and Ta_3_N_5_ shows that the EPD method can accommodate inorganic materials with different particle characteristics, surface chemistry, and electrode functions, provided that deposition voltage and time are adjusted accordingly.

### Metal chalcogenides

Layered chalcogenide materials, including MoS_2_ and WS_2_, were also successfully processed *via* EPD, demonstrating the applicability of this method to 2D semiconductors with distinct surface chemistry and electronic properties. Initial experiments in acetone without a charging agent gave only partial deposition on the positively biased electrode at 60 V after 180 s (Fig. S12a and b), indicating that the as-received MoS_2_ and WS_2_ particles possess weak intrinsic negative surface charge.

The addition of I_2_, which was effective for metal oxide and carbon-based systems, resulted in rapid aggregation and precipitation of both MoS_2_ and WS_2_, preventing stable suspension formation. This behaviour suggests that I_2_ disrupts the surface charge balance in chalcogenide systems, reducing their colloidal stability. Therefore, 2.5 mM citric acid was used as an alternative charging agent. Its carboxylate groups can adsorb onto layered chalcogenide surfaces, imparting negative charge and improving electrostatic stabilisation. Although other carboxylate-based additives could also be explored,^30^ citric acid provided a simple and effective route to stable MoS_2_ and WS_2_ suspensions, enabling reproducible anodic deposition.

**Fig. 5:**
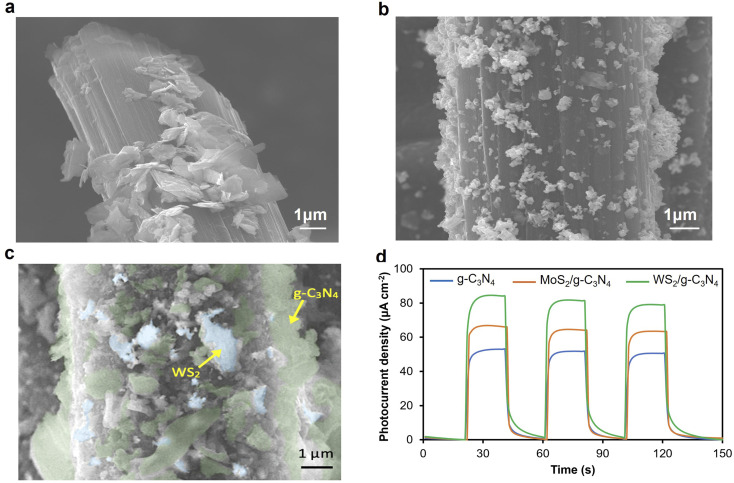
EPD of layered metal chalcogenides and their integration into g-C_3_N_4_ hybrid photoelectrodes. SEM images of (a) MoS_2_, (b) WS_2_ and (c) WS_2_/g-C_3_N_4_ deposited on carbon paper. (d) Photocurrent responses of CP/g-C_3_N_4_, CP/MoS_2_/g-C_3_N_4_ and CP/WS_2_/g-C_3_N_4_ electrodes measured in 0.5 M KHCO_3_ containing 10% triethanolamine under simulated solar irradiation. The enhanced photocurrent of WS_2_/g-C_3_N_4_ indicates improved interfacial charge extraction.

Using citric acid-stabilised suspensions (1 mg mL^−1^), uniform and adherent films of MoS_2_ (60 V for 90 s) and WS_2_ (60 V for 60 s) were successfully deposited on both ITO (Fig. S12c and d) and carbon paper. SEM analysis confirms successful deposition of layered chalcogenide materials. MoS_2_-coated electrode exhibits flake-like particles on the carbon fibres (Fig. 5a), but appears relatively discontinuous, with larger agglomerated flakes distributed across the surface. In contrast, the WS_2_-coated film shows a more uniform distribution of smaller particles (Fig. 5b), indicating improved surface coverage and better interfacial contact under the optimised conditions.

To evaluate their function as electron-extraction layers, g-C_3_N_4_ was subsequently deposited by EPD onto MoS_2_/CP and WS_2_/CP substrates using the optimised conditions. The resulting bilayer photoelectrodes showed enhanced anodic photocurrent responses compared with the g-C_3_N_4_-only electrode, indicating improved charge separation and electron transport. The SEM image of the CP/WS_2_/g-C_3_N_4_ electrode confirms the successful integration of WS_2_ and g-C_3_N_4_ on the carbon paper fibre (Fig. 5c). This architecture is expected to facilitate electron transfer from g-C_3_N_4_ to the current collector and suppress charge recombination. Among the two chalcogenide interlayers, WS_2_ exhibited superior performance compared with MoS_2_. The photocurrent response of g-C_3_N_4_ increased by 1.6-fold in the presence of the WS_2_ layer, whereas a 1.2-fold enhancement was observed with MoS_2_ (Fig. 5d). This difference is attributed to the more uniform WS_2_ coverage and more effective interfacial charge extraction from g-C_3_N_4_. It indicates that WS_2_ functions as a more efficient electron-extraction layer under the optimised EPD conditions.

Collectively, these findings demonstrate that EPD can be adapted to materials with different intrinsic charging behaviours through appropriate selection of charging agents. The successful deposition of MoS_2_ and WS_2_, together with their integration into g-C_3_N_4_-based hybrid photoelectrodes, highlights the versatility of EPD for photoelectrochemical applications.

### MOF and COF materials

The versatility of the EPD platform was further demonstrated using porous framework materials, including MOF, COF, and COF/carbon nanostructure composites. Framework materials are often difficult to process into uniform electrode films because of their low density, limited electrical conductivity, and tendency to form poorly adherent coatings. Despite these challenges, EPD enabled their direct assembly into continuous films on conductive substrates, demonstrating the broader applicability of this method beyond conventional inorganic and carbon-based materials.

**Fig. 6:**
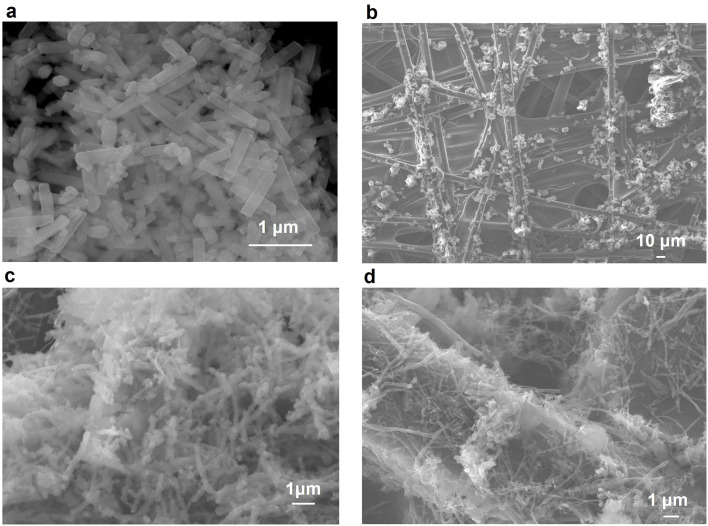
EPD of porous framework and framework–carbon composite electrodes. SEM images of (a) NU-1000 MOF deposited on ITO, (b) COF-366-Co deposited on carbon paper, (c) COF-366-Co/GNF composite deposited on carbon paper and (d) COF-366-Co/MWCNT composite deposited on carbon paper. The images demonstrate that EPD can assemble both porous framework particles and conductive framework–carbon composites into adherent electrode coatings.

NU-1000 MOF was first examined as a representative porous framework material. NU-1000 was deposited on ITO from an acetone + I_2_ suspension at 10 V for 90 s (Fig. S13a). The SEM image of the EPD-coated NU-1000 film shows well-defined rod-like crystallites with preserved morphology (Fig. 6a), indicating that the deposition process does not significantly damage the framework structure. This confirms that EPD can assemble fragile anisotropic MOF crystallites into adherent electrode coatings while preserving their framework morphology.

The method was then extended to COF-based materials and their composites with carbon nanostructures, which are of particular interest for electrocatalytic CO_2_ reduction. COF-366-Co was deposited from a 0.3 mg mL^−1^ suspension in acetone without any additional charging agent. Interestingly, deposition occurred on the positively biased CP electrode at 30 V for 3 min (Fig. S13b), indicating that the synthesised COF possesses sufficient intrinsic negative surface charge for electrophoretic migration. SEM image shows COF particles distributed along the carbon fibre and fibre junctions, while the open macroporous structure of carbon paper remains visible (Fig. 6b). This partial but conformal coating is advantageous for electrolyte penetration and catalytic sites exposure, although conductive carbon additives are required to improve electrical connectivity.

COF-366-Co/GNF composite was successfully deposited on carbon paper from an acetone + I_2_ suspension with a concentration of 0.083 mg mL^−1^ under 60 V for 1 min (Fig. S13c). The resulting film reveals an interconnected porous coating in which the GNF network is integrated with COF particles, providing close physical contact between the framework and conductive carbon component (Fig. 6c). Similarly, the COF-366-Co/MWCNT composite was EPD-coated on carbon paper from a 0.25 mg mL^−1^ suspension in acetone + I_2_ under 60 V for 5 min (Fig. S13d). The COF-366-Co/MWCNT electrode exhibits a fibrous nanotube network containing distributed COF particles, providing electron-transport pathways while maintaining electrolyte-accessible porous regions (Fig. 6d).

Overall, the successful deposition of NU-1000 MOF, COF-366-Co, COF-366-Co/GNF, and COF-366-Co/MWCNT systems demonstrates that EPD is a broadly applicable strategy for integrating porous framework materials into functional electrodes. Importantly, the method accommodates materials with different intrinsic charging behaviours, from intrinsically negatively charged COF particles deposited without additives to framework–carbon composites requiring I_2_-assisted suspension stabilisation. This adaptability further supports the applicability of EPD for scalable fabrication of diverse photoelectrochemical and electrocatalytic electrode architectures.

### General relationships between material chemistry, suspension stability and EPD parameters

Across the different material classes, the optimised EPD parameters were governed by the combined effects of surface chemistry, particle morphology, density, conductivity, suspension stability, and substrate architecture. A common feature of the successful depositions was the use of acetone-based suspensions, highlighting the suitability of acetone as a versatile low-viscosity, volatile, and weakly polar medium for EPD of diverse particulate materials. However, acetone alone was not sufficient for most systems; material-specific charging agents were required to generate or stabilise surface charge. Materials with low native surface charge, such as carbon black, required additive-assisted charging using I_2_ and water to generate sufficient electrophoretic mobility and uniform deposition. High-aspect-ratio carbon materials, including MWCNTs and GNFs, required surface oxidation to introduce oxygen-containing functional groups, improve dispersion stability, and enable interconnected conductive network formation on carbon paper. Relatively small oxide particles, such as TiO_2_, formed compact coatings at low voltage when sufficient surface charge was generated, whereas denser inorganic particles, such as WO_3_ and Ta_3_N_5_, required higher voltage and/or longer deposition time to achieve adequate film coverage. Metal chalcogenides, including MoS_2_ and WS_2_, were destabilised by I_2_ but stabilised by citric acid, indicating that surface chemistry and aggregation tendency control charging-agent selection, suspension stability, and deposition polarity. Furthermore, I_2_ may chemically react with these materials, forming metal iodides with bridging iodine anions, crosslinking the particles and decreasing their stability in the suspension. Porous framework materials also showed distinct behaviour depending on intrinsic charge and conductivity: COF-366-Co deposited without a charging agent because of its negative surface charge, whereas COF/carbon composites required I_2_-assisted charging and benefited from conductive carbon networks. These trends show that successful EPD requires matching the solvent/additive system and deposition parameters to the particle size, shape, density, surface chemistry, conductivity, and target electrode function, rather than applying a single universal deposition condition.

The substrate architecture also strongly influenced EPD behaviour and the choice of deposition conditions, as summarised in Table S1. Planar ITO was useful for producing compact and visually uniform films, particularly for optical measurements, and photoelectrode evaluation where a flat transparent conductive substrate is required. In contrast, porous carbon paper was more beneficial for carbon nanostructures, carbon nitride photoelectrodes, chalcogenide interlayers, oxide supports, and MOF/COF–carbon composites because its fibre network provides high surface area, electrolyte accessibility, and conductive pathways. However, deposition on porous substrates requires careful control of voltage and time. Short deposition times or low voltages can result in incomplete fibre coverage, whereas excessive voltage or prolonged deposition can generate thick coatings, block macropores, reduce electrolyte access, and increase charge-transport resistance. Therefore, the optimum EPD condition depends not only on material surface chemistry and particle morphology, but also on substrate architecture and the intended electrode function.

Adhesion behaviour of binder-free EPD-coated films was evaluated using tape-peel tests on representative Ta_3_N_5_ and g-C_3_N_4_ electrodes deposited on both ITO and carbon paper substrates. The EPD-coated Ta_3_N_5_ film on planar ITO showed significant film removal after tape peeling, indicating weaker mechanical retention (15%) on the flat substrate (Fig. S14 and Table S2). In contrast, the corresponding film on carbon paper retained 87% of the deposited coating, with only minor material loss. This improved adhesion is attributed to conformal deposition around the porous carbon fibre network, which increases substrate–film contact area and provides mechanical interlocking. Similar behaviour was observed for g-C_3_N_4_ electrodes (Table S2). These results confirm that binder-free EPD produces films with sufficient adhesion for handling and photoelectrochemical testing, particularly on porous carbon paper substrates, while also highlighting that planar substrates may require further surface modification or post-treatment when stronger mechanical adhesion is required.

Long-term chopped-light photocurrent measurement was conducted to evaluate the operational stability of the EPD-coated photoelectrode. As a representative example, the Ta_3_N_5_/CP electrode was tested for 5 h under repeated light on/off illumination cycles (Fig. S15). The photocurrent response remained stable over the measurement period, with only a 13% decrease in photocurrent after 5 h, corresponding to approximately 87% retention of the initial activity. After the stability test, the electrode was gently washed, dried, and re-tested under the same conditions. The recovered photocurrent was comparable to the initial response, indicating that the observed decrease during prolonged operation is largely reversible. This behaviour suggests that the small photocurrent loss is more likely associated with temporary surface blocking, adsorption of electrolyte-derived species, or gas-bubble accumulation during operation, rather than irreversible film detachment or severe degradation of the EPD-coated Ta_3_N_5_ layer. These results further support the stability and practical robustness of the binder-free EPD-coated photoelectrode.

Although SEM and TEM confirm film formation and substrate coverage, detailed quantitative measurements of film thickness, surface roughness, and porosity were not performed for every material system. In the present broad material-screening study, film loading, deposition time, applied voltage, deposition polarity, and morphology from SEM/TEM were used as practical descriptors of EPD behaviour. This approach is particularly relevant for porous carbon paper substrates, where the deposited material coats and penetrates the three-dimensional fibre network, making a single film-thickness value difficult to define. Therefore, the present results establish material compatibility, coating morphology, and functional electrode behaviour, while detailed cross-sectional thickness, roughness, and porosity analysis will be important for future optimisation of selected electrode systems.

### Applicability and limitations of the EPD

The broad applicability of this EPD platform makes it relevant beyond making electrodes for electrocatalysis and photoelectrocatalysis. For example, EPD has been used to fabricate lithium-ion battery electrodes, including commercial active-material electrodes and large-area electrodes for pouch-cell configurations.^31^ It has also been applied to supercapacitor electrodes, such as MnO_2_/graphene, nickel–cobalt hydroxide/graphene, and NiCo-LDH-based binder-free electrodes.^32,33^ In sensor applications, EPD has been used to fabricate TiO_2_-based NO_2_ gas sensors, SnO_2_/graphene oxide VOC sensors, and graphene oxide/MoS_2_-coated quartz crystal microbalance sensors.^34–36^ These examples show that EPD is a transferable electrode-fabrication method for energy storage, electrocatalysis, sensing, and photoelectrochemical systems. However, the approach is not universal in the sense of using a single recipe for all materials. Successful EPD requires each material to form a stable charged suspension, exhibit sufficient electrophoretic mobility, and deposit onto a conductive substrate without excessive aggregation, cracking, or pore blockage. Therefore, solvent, charging agent, deposition polarity, voltage, time, substrate architecture, and post-treatment must be optimised according to the target material and application.

To further evaluate the practical advantage of EPD over conventional coating approaches, g-C_3_N_4_ films were prepared on ITO by drop-casting and spin-coating under representative conditions. The drop-cast film showed patchy and non-uniform coverage even after three successive ink depositions, while the spin-coated film showed material accumulation near the electrode edges and reduced coverage in the central region (Fig. S16a). In contrast, EPD produced a more uniform and adherent g-C_3_N_4_ film under the optimised conditions. Photocurrent measurements further showed that the drop-cast and spin-coated films exhibited approximately seven-fold and ten-fold lower photocurrent responses, respectively, compared with the EPD-coated g-C_3_N_4_ film (Fig. S16b). These results confirm that, for the representative g-C_3_N_4_ system, EPD provides improved film uniformity and functional photoelectrochemical performance compared with simple drop-casting and spin-coating.

EPD offers several advantages over conventional coating methods. The EPD process is carried out at room temperature over short deposition times and does not require polymeric binders, high-temperature precursor growth, or vacuum-based instrumentation. The energy input for EPD is mainly associated with applying an electric field across the suspension and can be estimated from the applied voltage, deposition current, and deposition time. However, the actual power consumption depends strongly on suspension conductivity, electrode spacing, electrode area, and bath resistance. In terms of material utilisation, EPD allows direct deposition of charged particles onto the target electrode, but the deposition efficiency is influenced by particle concentration, electrophoretic mobility, suspension stability, and deposition geometry. Undeposited material remaining in the suspension could potentially be reused, although bath ageing, aggregation, and additive concentration must be controlled for reproducible processing. Compared with vacuum deposition and hydrothermal growth, EPD offers simpler instrumentation, lower processing complexity, and compatibility with both planar and porous substrates. Compared with slurry-based coating methods, it avoids inactive binders and can provide conformal coatings on three-dimensional conductive supports. Nevertheless, economic feasibility at large scale will require optimisation of bath lifetime, solvent recovery, deposition uniformity over large areas, material utilisation efficiency, and post-treatment requirements. Thus, EPD should be considered a practically attractive and scalable fabrication platform, while further process-level analysis is needed for application-specific scale-up.

### Scalability considerations

EPD is a potentially scalable and scale-up-compatible coating method because it is solution-processable, binder-free, electrically driven, and compatible with both planar and porous conductive substrates. In the present study, EPD coating was demonstrated primarily using 1 cm^2^ electrodes and further validated using a larger 2.5 cm^2^ g-C_3_N_4_-coated carbon paper and FTO electrodes prepared under the optimised conditions (Fig. S17). This provides preliminary evidence that the deposition process can be conveniently scaled up by simply increasing the surface area of the electrode without compromising film uniformity. In principle, further extension to larger electrode areas, such as 5, 10, or 20 cm^2^, should be feasible by increasing the bath size while maintaining uniform electrode spacing, suspension stability, electric-field distribution, and controlled deposition time. However, continuous deposition, bath reuse, solvent recovery, and large-area process reproducibility will require dedicated scale-up studies, which are beyond the scope of the present work.

## Conclusion

This work successfully demonstrated electrophoretic deposition as a versatile, binder-free, and scalable platform for converting diverse functional powders and particulate materials into uniform electrode films. By tailoring suspension chemistry, charging agents, voltage, and deposition time, EPD was successfully adapted to conductive carbons, carbon nitrides, metal oxides, nitrides, chalcogenides, MOF/COF, and hybrid composites on both ITO and carbon paper substrates. The results show that effective EPD requires a balance between particle charging, suspension stability, and controlled film growth. Iodine-assisted acetone suspensions were suitable for many systems, while citric acid enabled stable deposition of layered chalcogenides. Photocurrent measurements further confirmed that optimised loading is essential for achieving functional photoelectrochemical performance. Overall, this study establishes EPD as a broadly applicable and adaptable platform for fabricating electrodes and photoelectrodes, while recognising that material-specific optimisation of suspension chemistry and deposition parameters is essential for achieving reproducible film formation.

## Author contributions

M. T. developed the methodology. M. T., T. M. L., E. C. K., S. H., P. K., and S. K. prepared materials. M. T. carried out EPD coating of all the materials while M. S. S. and S. H. specifically carried out EPD-coating of chalcogenides and COF-based materials, respectively. M. T. performed scanning electron microscopy imaging as well as electrochemical and photoelectrochemical experiments, while E. C. K. performed HRTEM. A. N. K., J. A. F., A. L. and A. E. L. secured funding and supervised the work. A. N. K. and M. T. oversaw the entire work. All authors contributed to writing the manuscript.

## Conflicts of interest

The authors declare that there are no conflicts of interest.

## Supplementary Material

RA-OLF-D6RA05969D-s001

## Data Availability

The data supporting the findings of this study are available within the article and its supplementary information (SI). Additional datasets generated during this study, including raw and processed data, are available from the corresponding author upon reasonable request. Supplementary information is available. See DOI: https://doi.org/10.1039/d6ra05969d.
