# Supplementary material for: Facile electrophoretic deposition of diverse functional materials for scalable electrode and photoelectrode fabrication
Source: RSC Adv. 2026 Aug 17;16(41):48310–24. doi: 10.1039/d6ra05969d (PMC13478889; doi:10.1039/d6ra05969d)
Supplement: Supplementary file 1 [file RA-016-D6RA05969D-s001.pdf]

Supplementary Information (SI) for RSC Advances.
This journal is © The Royal Society of Chemistry 2026

Facile Electrophoretic Deposition of Diverse Functional Materials for Scalable Electrode and Photoelectrode Fabrication

Madasamy Thangamuthu[a]*, Tara M. Lemercier[a], Emerson C. Kohlrausch[a], Sean Hoggett[a], Matthew S. Sandhu[a], Pekka Korhonen[a†], Andrea Laybourn[b], Anabel E. Lanterna[a], Jesum Alves Fernandes[a], Andrei N. Khlobystov[a]*

[a]School of Chemistry, University of Nottingham, University Park, Nottingham NG7 2RD, United Kingdom

[b]Institute of Process Research and Development & School of Chemistry, University of Leeds, Leeds, United Kingdom

†Deceased author

*madasamy.thangamuthu1@nottingham.ac.uk
*andrei.khlobystov@nottingham.ac.uk

Supporting information

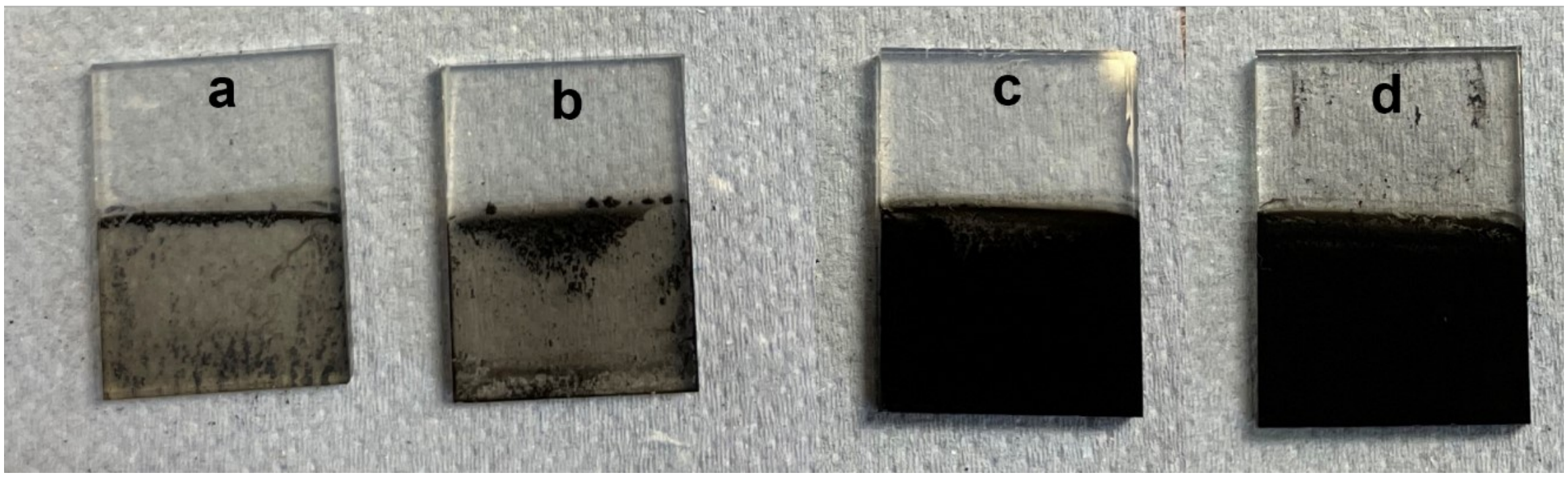

Fig. S1. Photographs of the EPD-coated film of carbon black (a-c) using the suspension prepared in a) acetone, b) acetone + I_2, c) 5 vol% water + acetone + I_2, and d) Pt-loaded carbon black deposited using 5 vol% water + acetone + I_2 suspension.

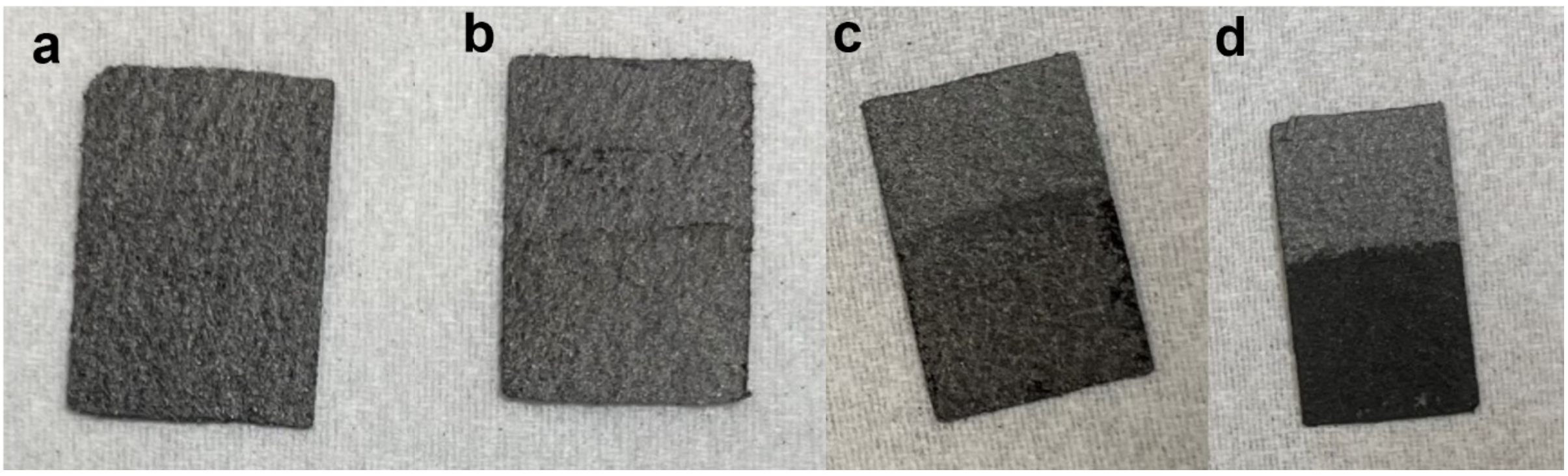

Fig. S2. Photographs of the EPD-coated film of MWCNT using the suspension prepared in a) ethanol + I_2, b) acetone + I_2, c) ethanol + acetone (1:1) + I_2, and d) oxidised MWCNT deposited using the suspension prepared in ethanol + acetone (1:1) + I_2.

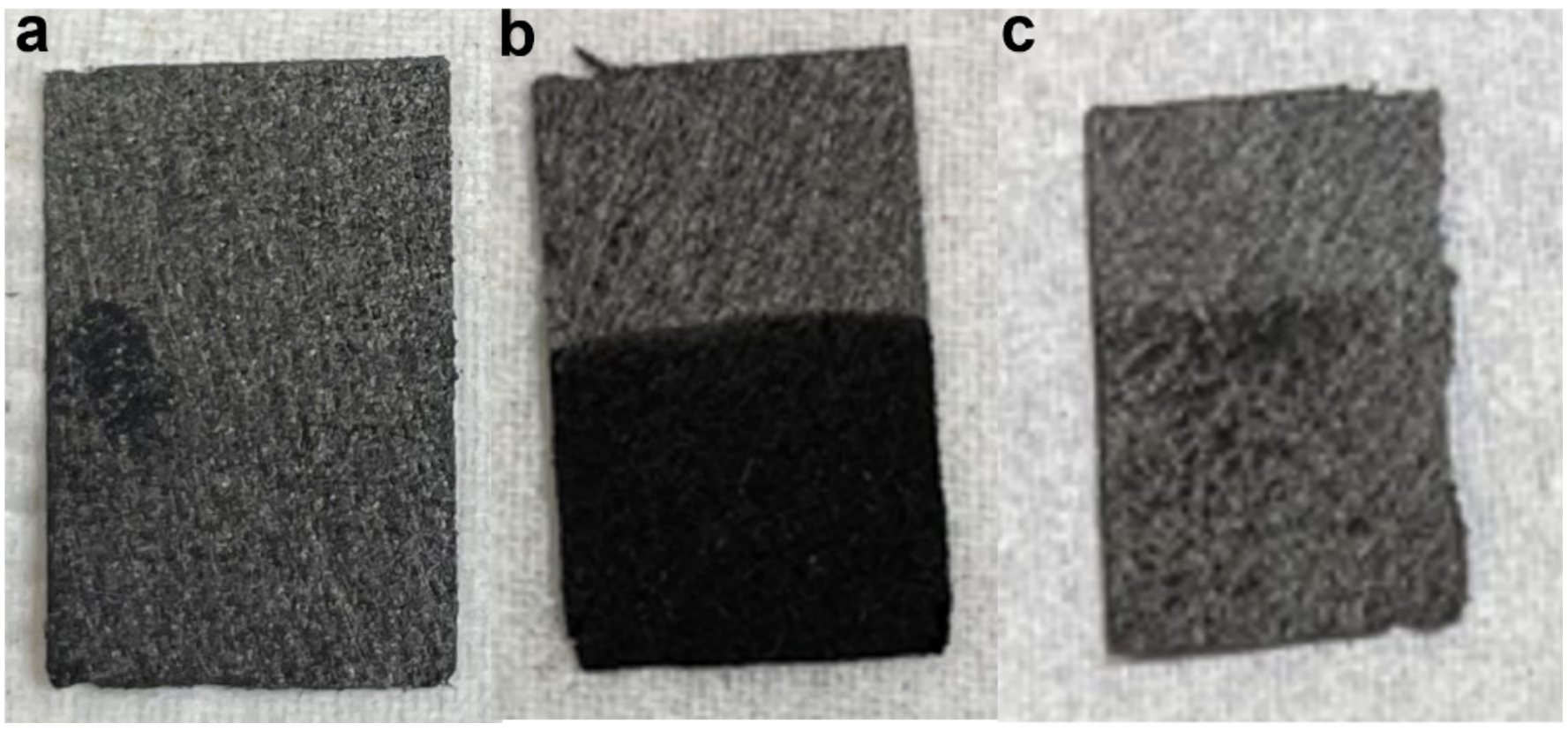

Fig. S3. Photographs of the EPD-coated film deposited from the suspension prepared in acetone + I_2 a) GNFs, b) oxidised GNFs, and c) oxidised GNFs deposited from the suspension prepared in ethanol + acetone (1:1) + I_2.

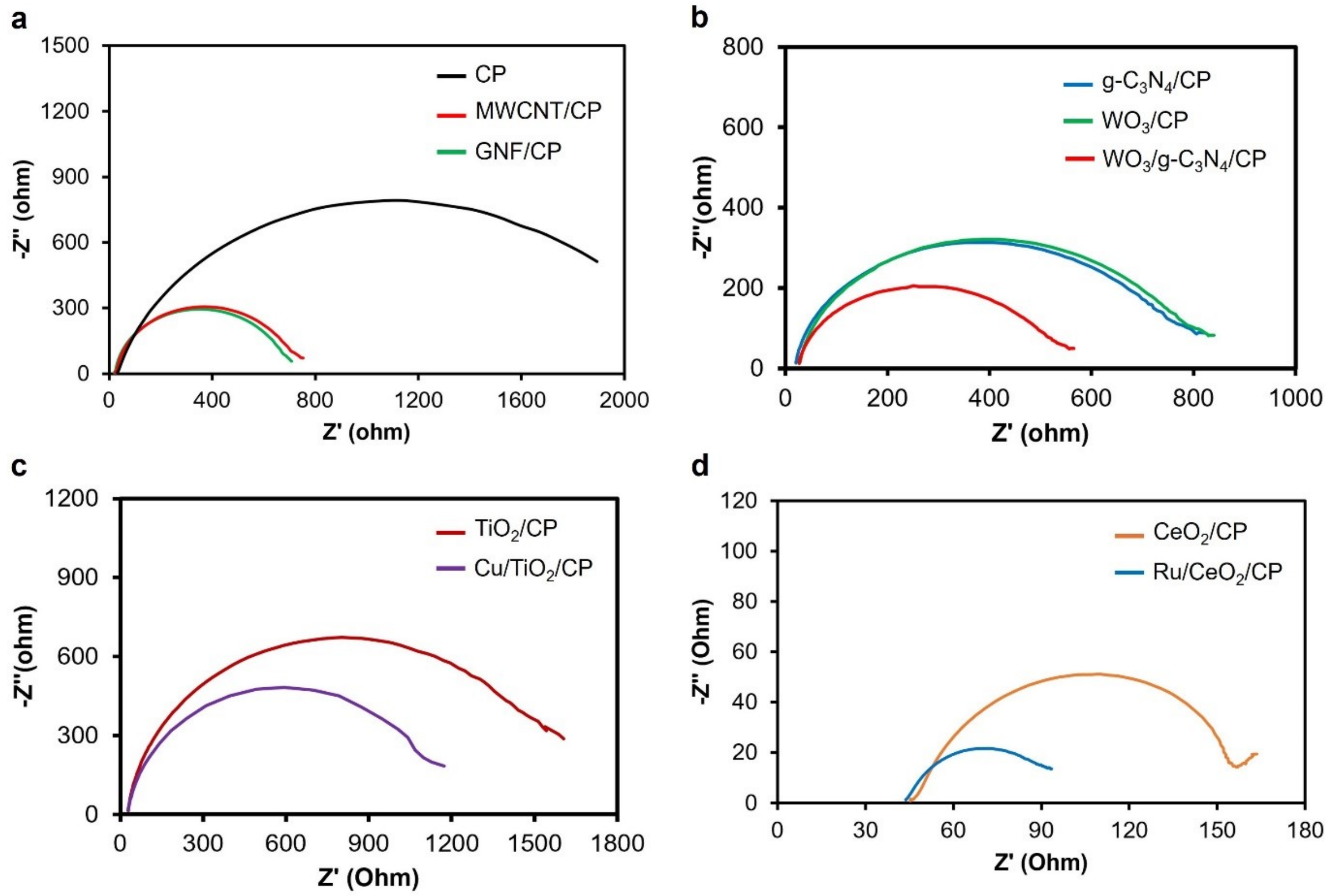

Fig. S4. Electrochemical impedance spectroscopy analysis of representative EPD-coated electrodes. Nyquist plots of (a) bare CP, MWCNT/CP and GNF/CP; (b) $g\text{-}C_3N_4$/CP, WO_3/CP and $WO_3/g\text{-}C_3N_4$/CP; (c) TiO_2/CP and Cu/TiO_2/CP; and (d) CeO_2/CP and Ru/CeO_2/CP. The reduced semicircle diameters observed for the conductive carbon-coated and hybrid electrodes indicate improved charge-transfer behaviour and effective electrical communication between the EPD-coated films and the conductive carbon paper substrate.

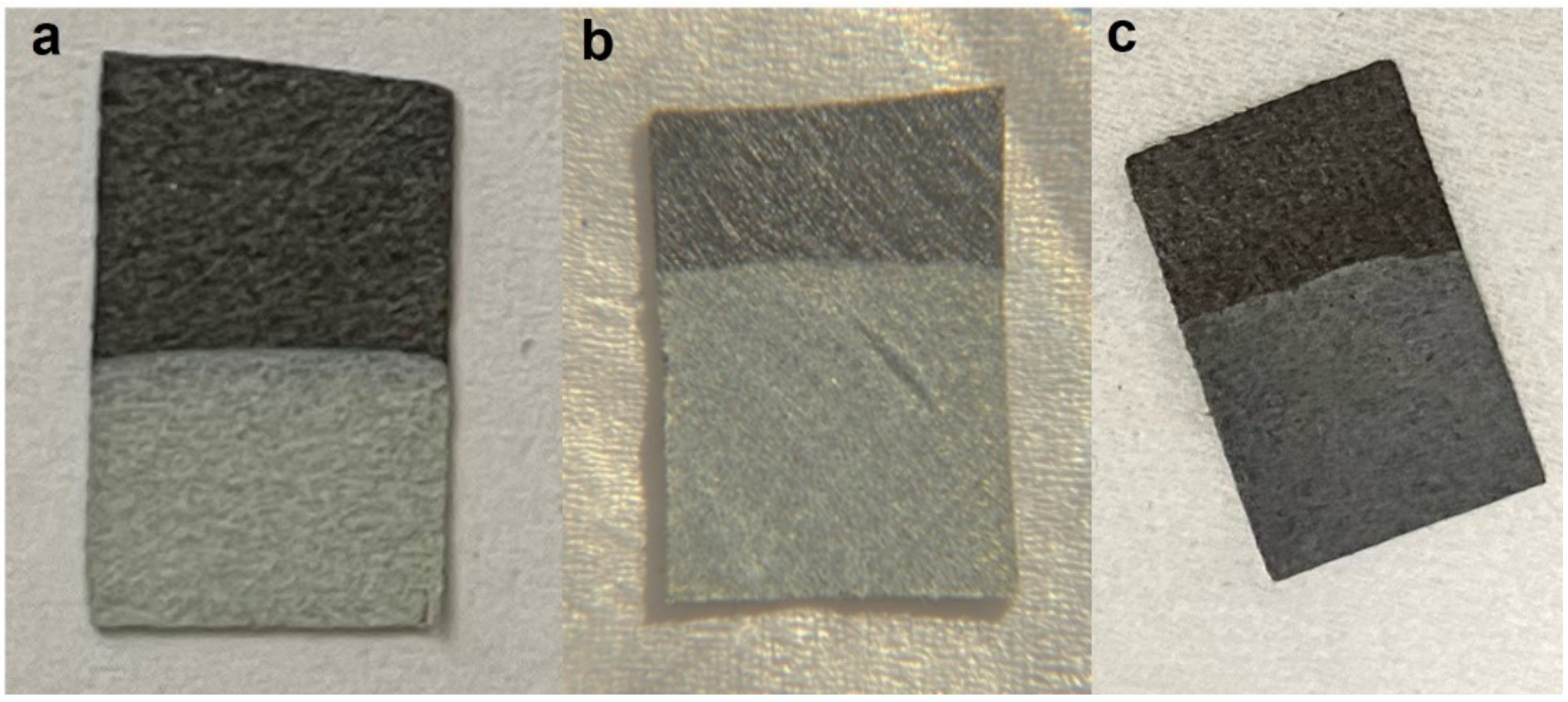

Fig. S5. Photographs of the EPD-coated film of a) g-C_3N_4, b) S-doped C_3N_4, and c) B-doped C_3N_4 deposited from their respective suspension prepared in acetone + I_2.

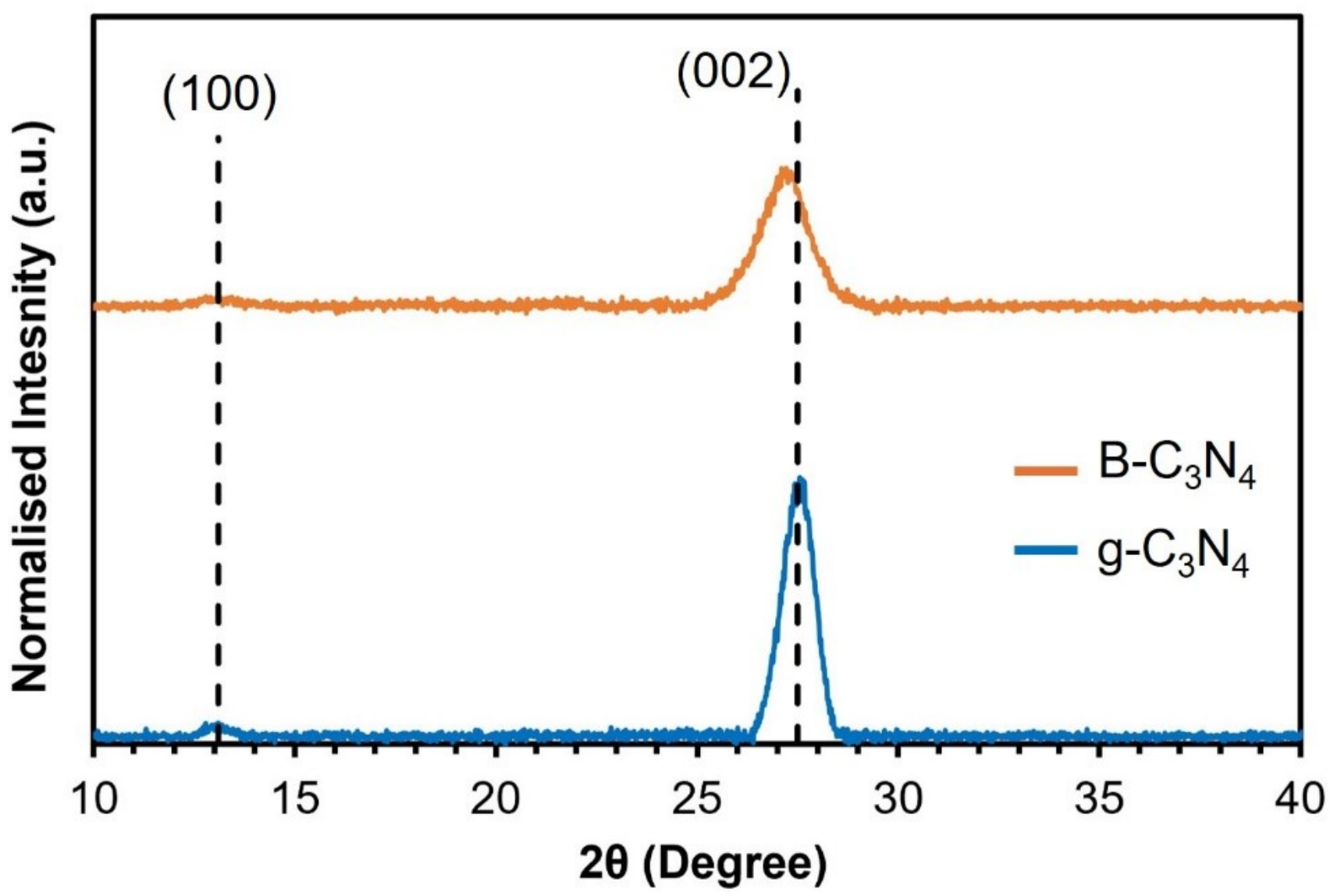

Fig. S6. XRD patterns of the g-C_3N_4 and B-doped C_3N_4

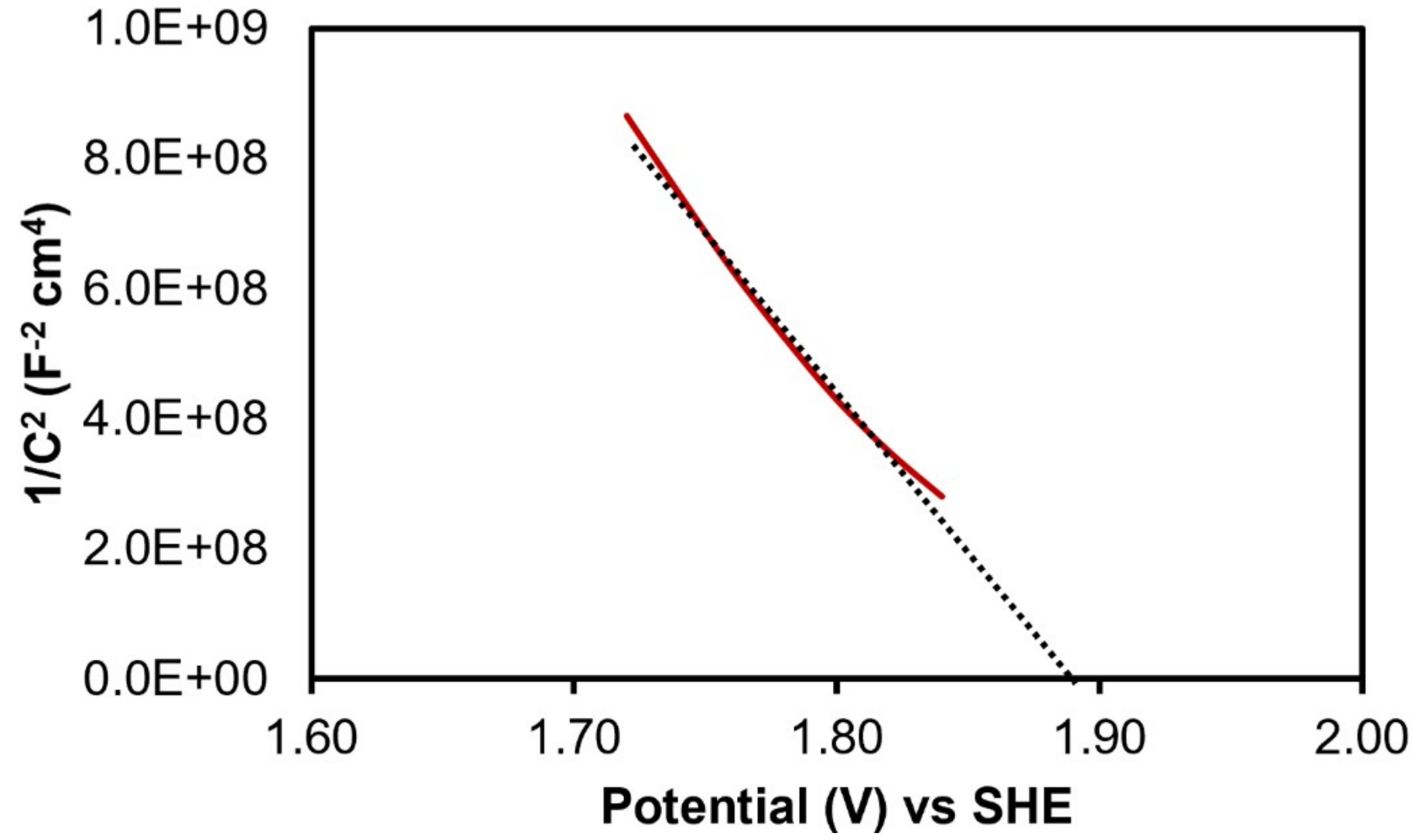

Fig. S7. Mott–Schottky plot of the EPD-deposited B-doped C_3N_4 thin film recorded in 0.1 M Na_2SO_4 electrolyte at a frequency of 1000 Hz. The negative slope confirms the p-type semiconducting nature of the material. The dashed line represents the linear fit used to determine the flat-band potential, yielding an intercept of approximately +1.9 V versus SHE.

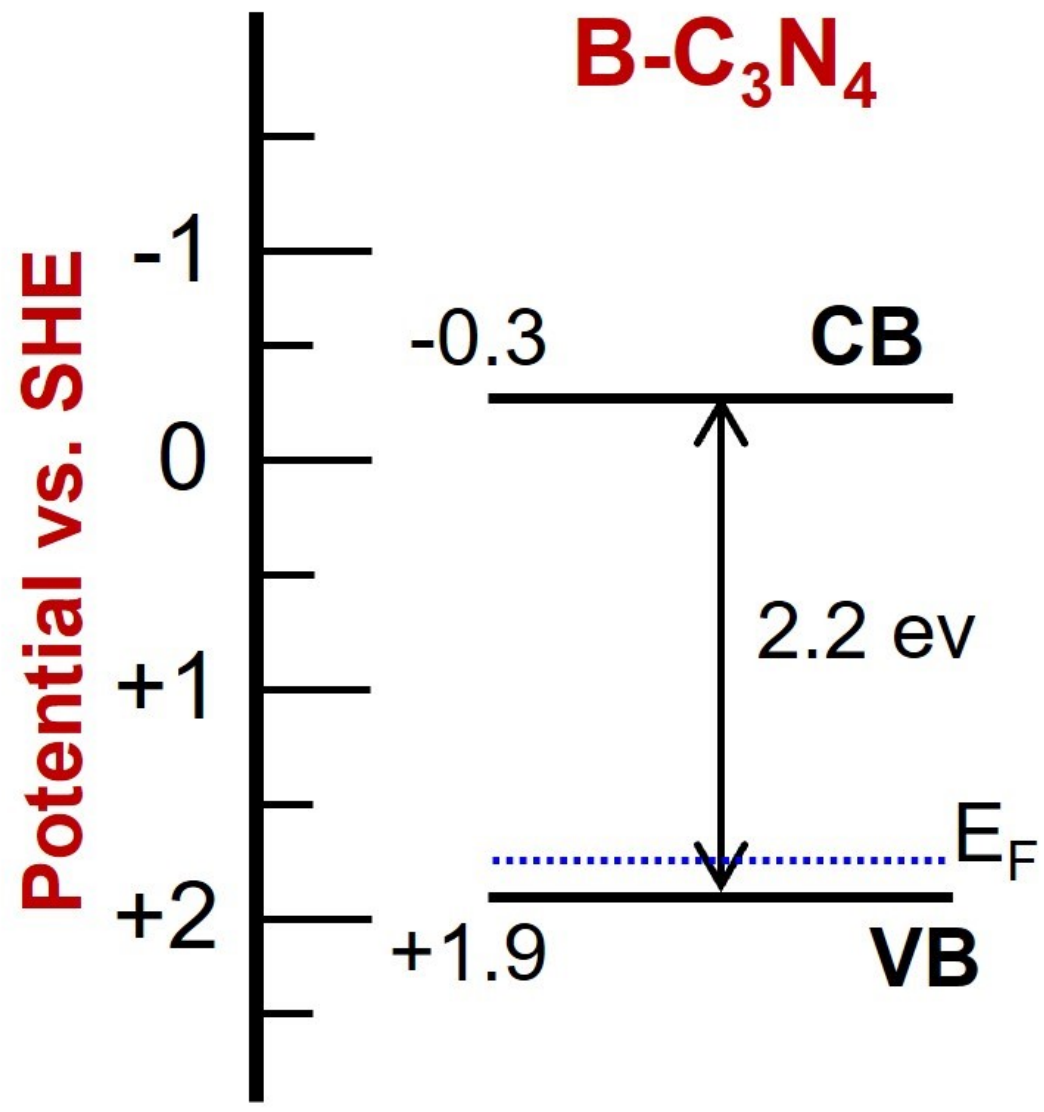

Fig. S8. Band-edge diagram of B-doped C_3N_4 constructed from the optical bandgap and Mott–Schottky analysis. The valence and conduction band edges were estimated to be approximately +1.9 and −0.3 V versus SHE, respectively.

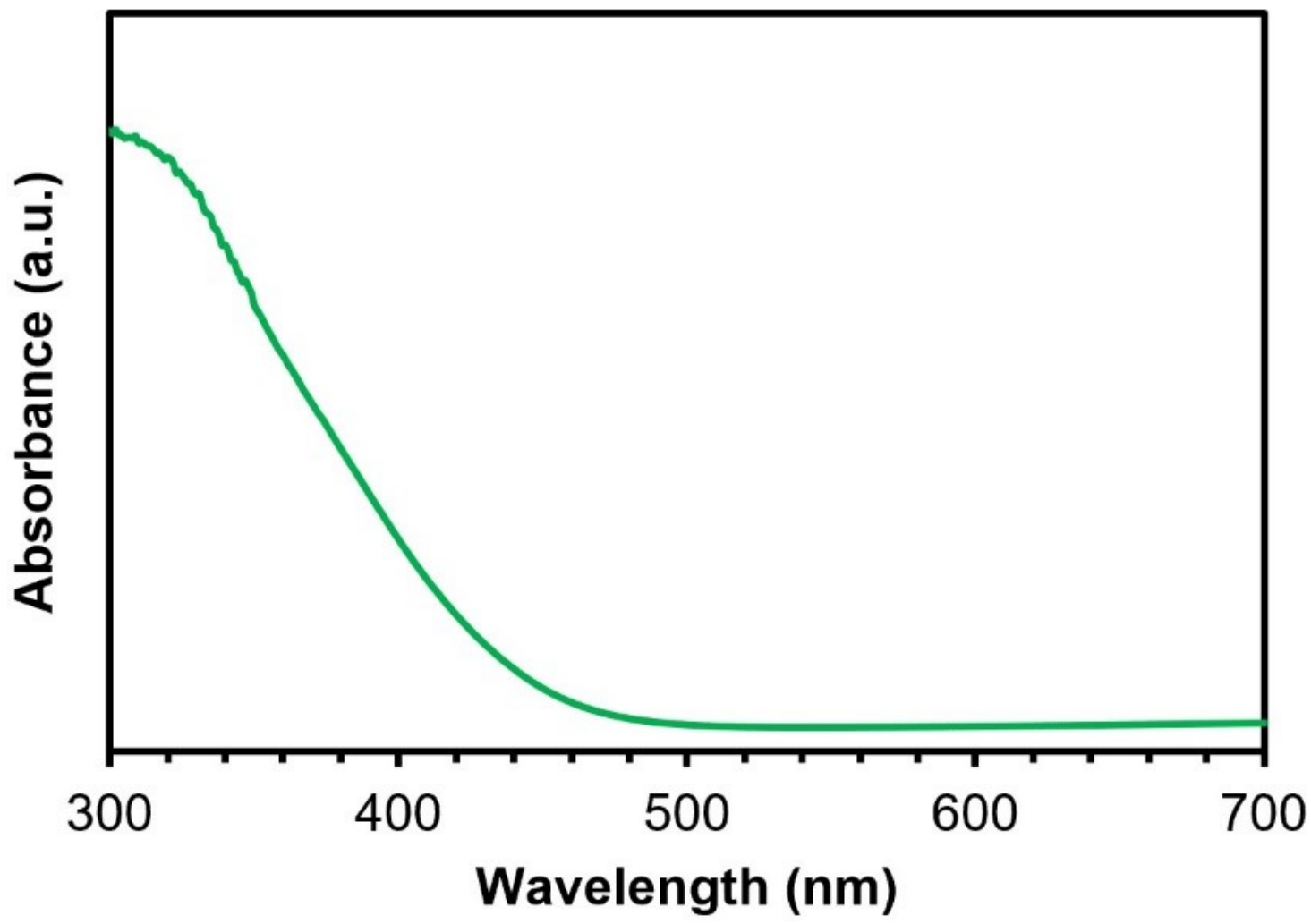

Fig. S9. UV-Vis. absorbance spectrum of the EPD-coated WO_3 thin film on an ITO electrode.

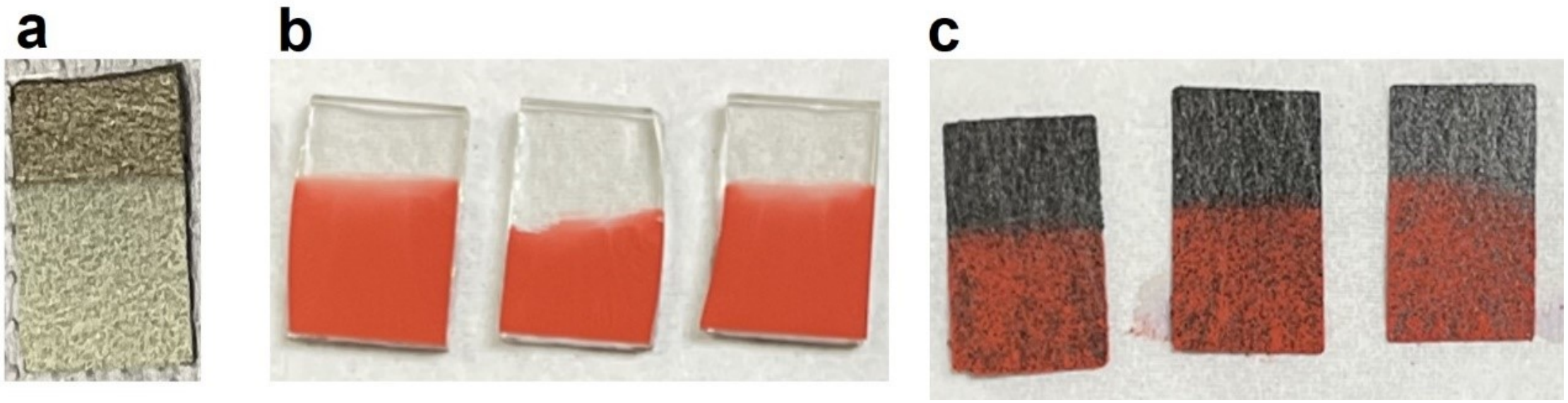

Fig. S10. Photograph of the EPD-coated films of a) CeO_2 thin film on a carbon paper electrode, Ta_3N_5 thin film on b) ITO and c) CP electrode.

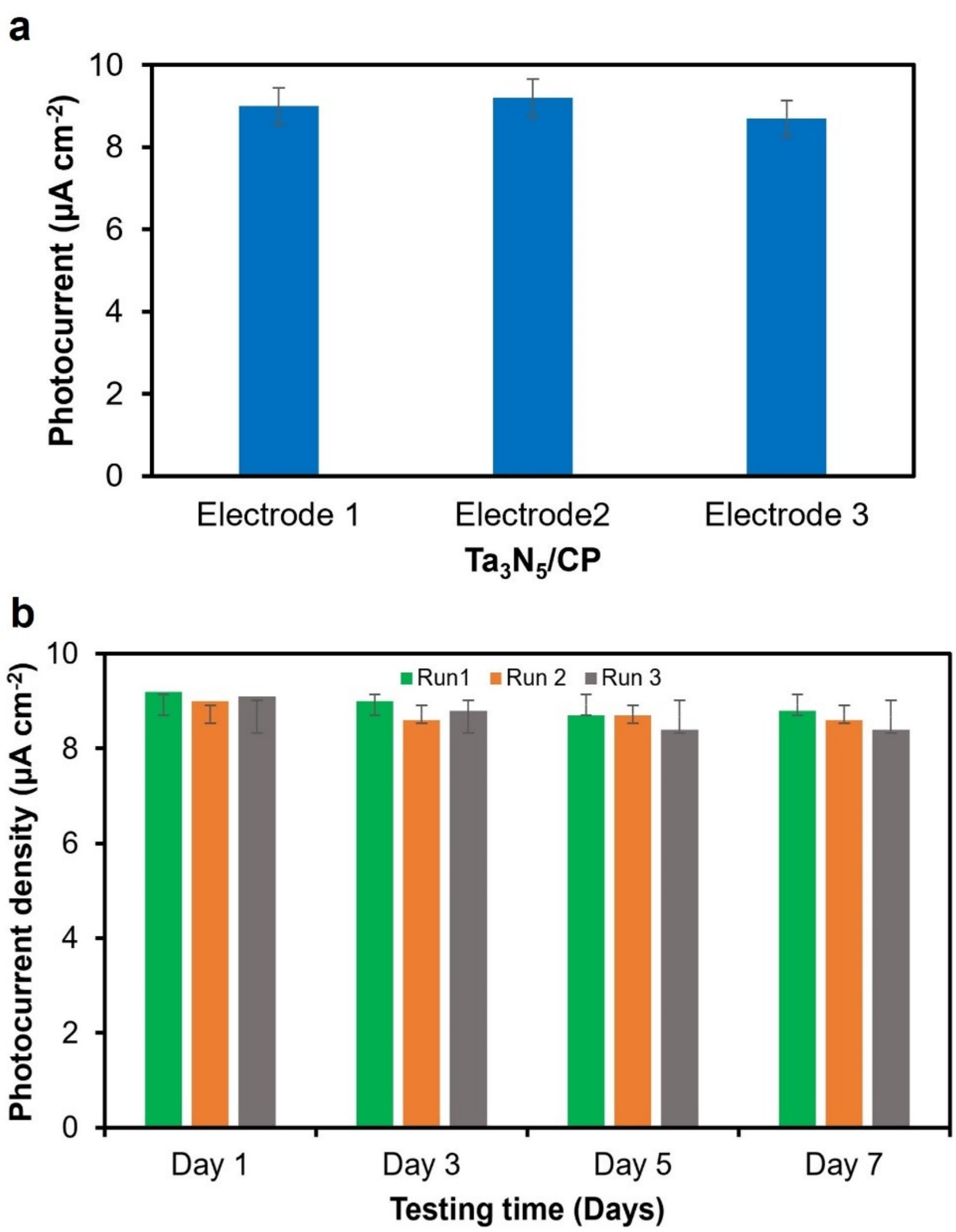

Fig. S11. Reproducibility and repeated-measurement stability of EPD-coated Ta_3N_5/CP photoelectrodes. (a) Photocurrent densities of three independently prepared Ta_3N_5/CP electrodes fabricated under identical EPD conditions, showing good electrode-to-electrode reproducibility. (b) Repeated photocurrent measurements of the same Ta_3N_5/CP electrode performed three times per day on day 1, day 3, day 5 and day 7, demonstrating stable photoelectrochemical response and good measurement repeatability. All photocurrent measurements were performed in 0.1 M KOH. Error bars represent the standard deviation from repeated measurements.

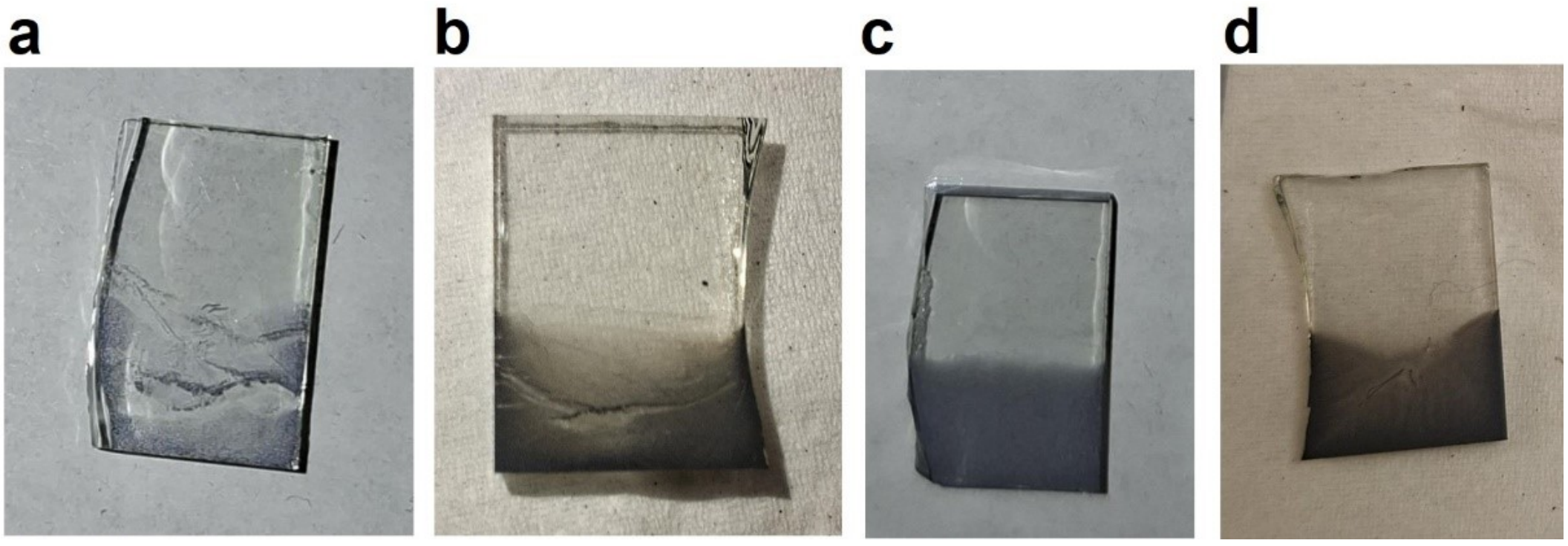

Fig. S12. Photographs of the EPD-coated (a) MoS_2, (b) WS_2 using their suspension prepared in acetone, and (c) MoS_2, (d) WS_2 using their suspension prepared in acetone + citric acid.

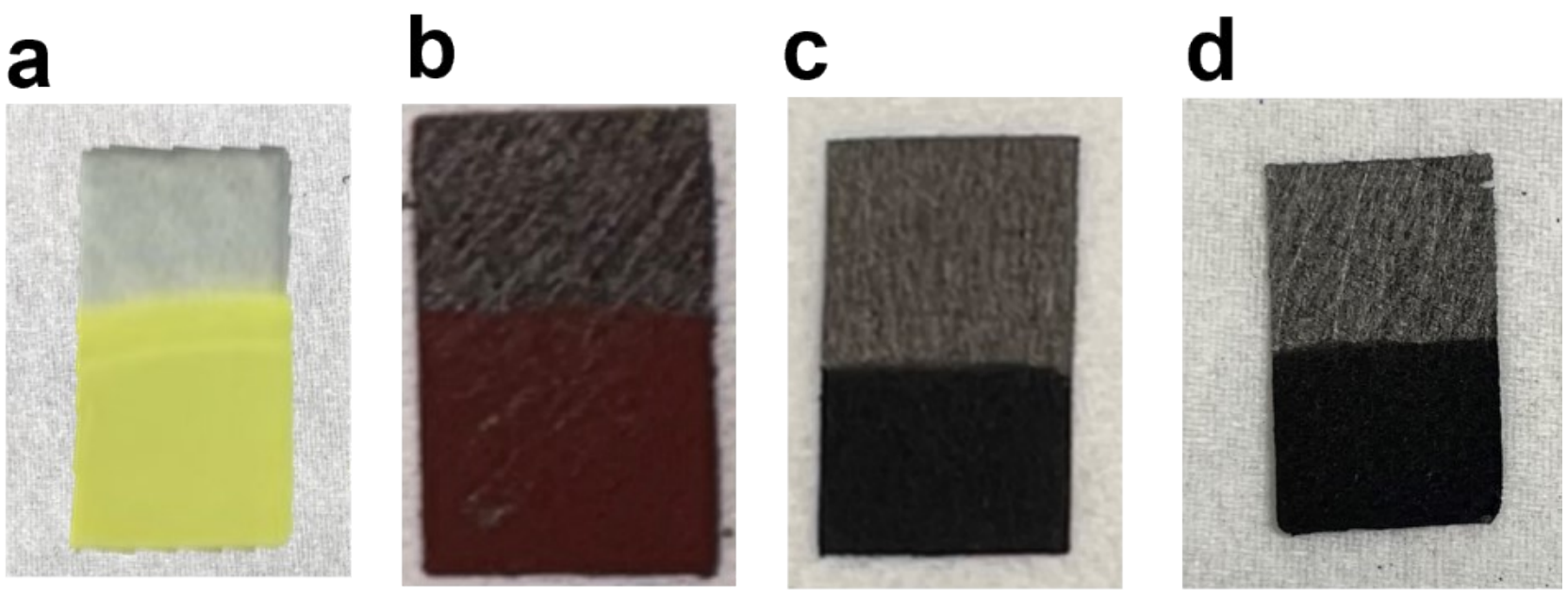

Fig. S13. Photographs of the EPD-coated (a) NU-1000 MOF, (b) COF-CoTBrPP-Bpin pyrene, (c) COF-366-Co/GNFs, and (d) COF-366-Co/MWCNTs.

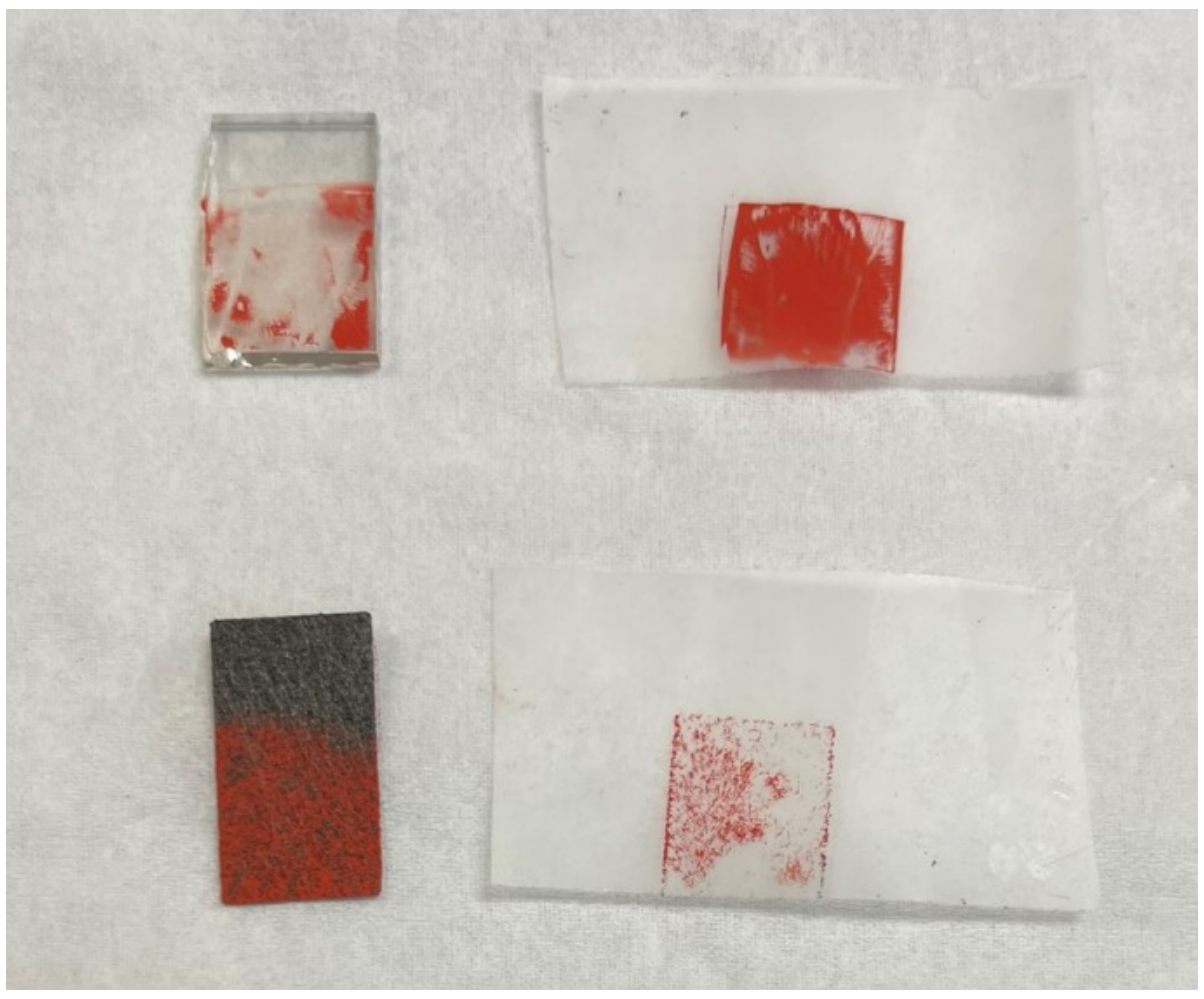

Fig. S14. Tape-peel adhesion assessment of representative binder-free EPD-coated electrodes. Optical photographs of Ta_3N_5-coated ITO and Ta_3N_5-coated carbon paper electrodes after tape-peel testing. The tape was applied to the coated area, gently pressed for 2 min, and peeled off under identical conditions. Significant film removal was observed from planar ITO, whereas only minor coating removal was observed from porous carbon paper, indicating stronger film retention on the fibre-based substrate.

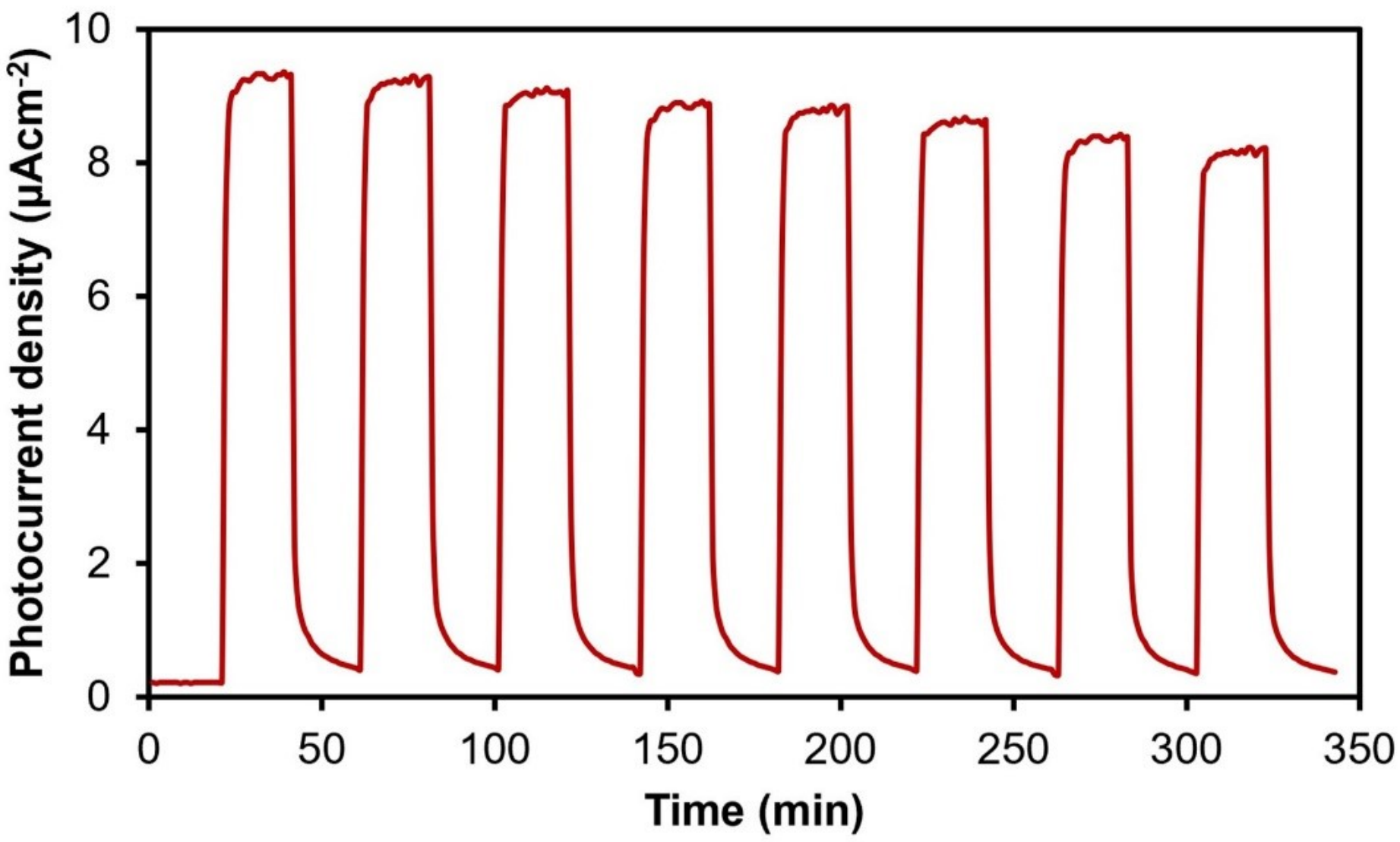

Fig. S15. Long-term photocurrent stability of the EPD-coated Ta_3N_5/CP photoelectrode. Chopped-light photocurrent response recorded over 5 h under repeated light on/off cycles in 0.1 M KOH solution at 1.23 V vs RHE.

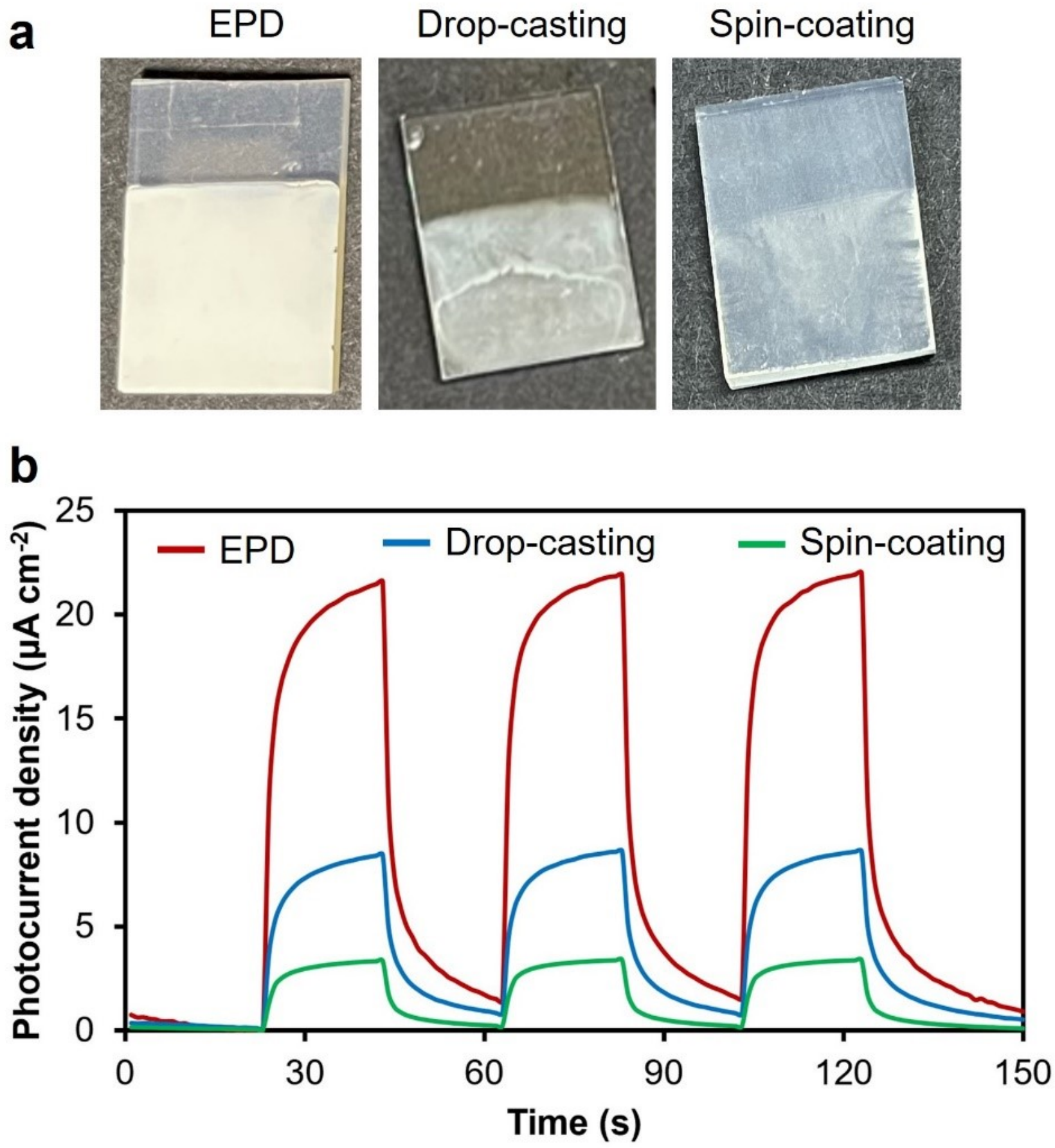

Fig. S16. Comparison of g-C_3N_4 film fabrication by EPD, drop-casting and spin-coating. (a) Photographs of g-C_3N_4 films prepared on ITO by EPD, drop-casting and spin-coating under representative conditions, (b) Chopped-light photocurrent responses of the corresponding g-C_3N_4 films measured in 0.1 M $KHCO_3$ solution under an argon atmosphere.

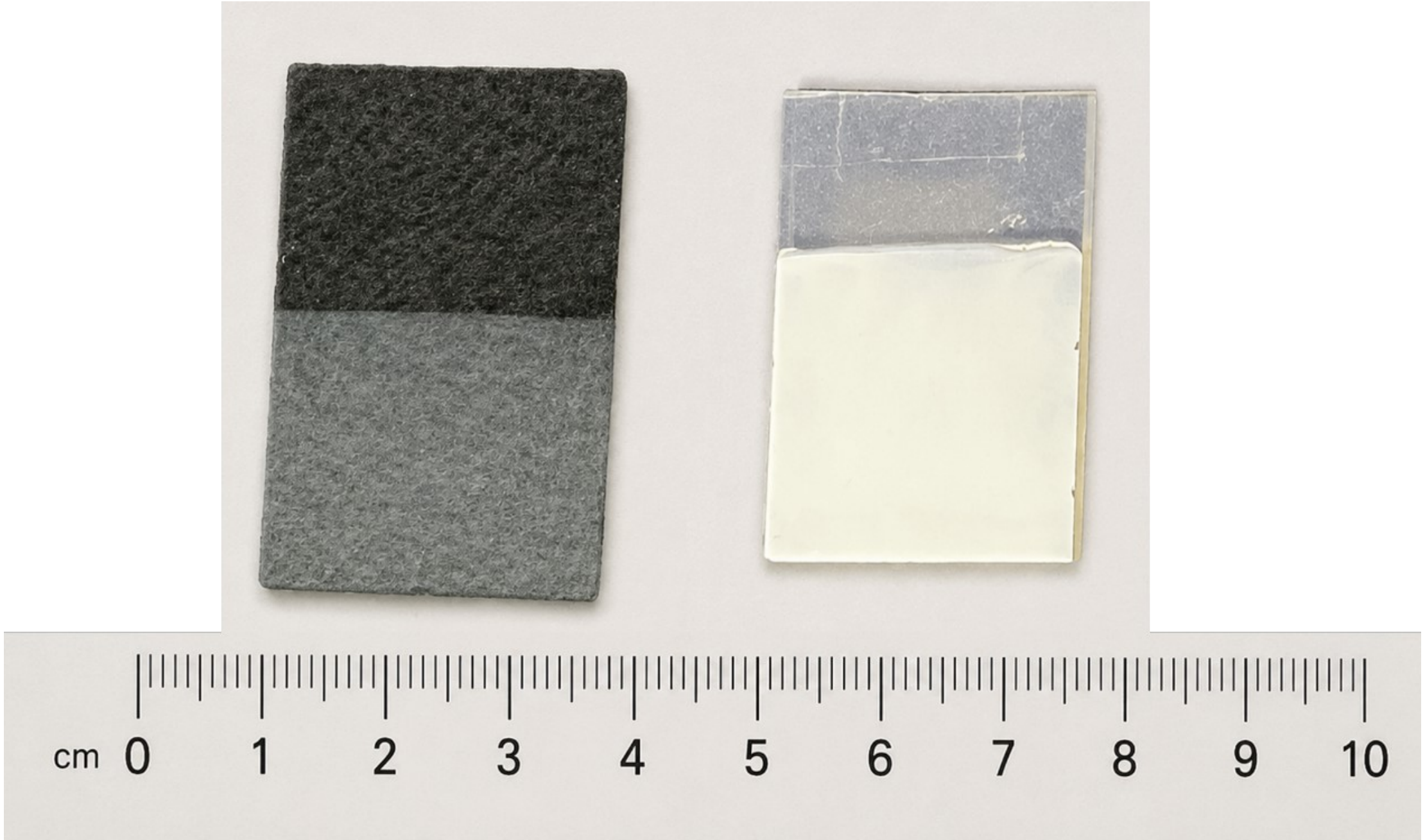

Fig. S17. Photograph of the EPD-coated g-C_3N_4 film on 2.5 cm^2 carbon paper and ITO substrates.

Table S1. Summary of optimised EPD conditions for each material system, including suspension composition, substrate type, deposition polarity, applied voltage, deposition time, and intended electrode function.

Material	Optimised Suspension	Substrate	Deposition electrode	Deposition voltage (V)	Deposition time (s)	Typical function
**Carbon-based conductors**						
Carbon black	0.5 mg mL^{-1} in acetone + I_2 + 5 vol% DI water	ITO	Cathode	60	300	Conductive scaffold/support for catalyst loading
Pt/carbon black	0.5 mg mL^{-1} in acetone + I_2 + 5 vol% DI water	ITO	Cathode	60	300	Direct electrocatalyst for electrochemical reactions
MWCNT	0.25 mg mL^{-1} in acetone + ethanol (1:1) + I_2	Carbon paper	Cathode	20	60	Conductive scaffold/support for catalyst loading
GNF	0.25 mg mL^{-1} in acetone + I_2	Carbon paper	Cathode	60	60	Conductive scaffold/support for catalyst loading
**Polymeric semiconductor (g-C_3N_4 family)**						
g-C_3N_4	2 mg mL^{-1} in acetone + I_2	Carbon paper	Cathode	60	30	Photoanode for water and organic oxidation reactions
Cu/g-C_3N_4	2 mg mL^{-1} in acetone + I_2	Carbon paper	Cathode	50	90	Photoanode for water and organic oxidation reactions
S-C_3N_4	3 mg mL^{-1} in acetone + I_2	Carbon paper	Cathode	50	120	Photoanode for water and organic oxidation reactions
B-C_3N_4	5 mg mL^{-1} in acetone + I_2	Carbon paper	Cathode	60	120	Photocathode for CO_2 reduction
**Metal oxides and nitrides**						
TiO_2 (Anatase)	0.2 mg mL^{-1} in acetone + I_2	ITO	Cathode	10	30	Stable photoanode for oxidation reaction
TiO_2 (P25)	0.2 mg mL^{-1} in acetone + I_2	ITO	Cathode	10	45	Stable photoanode for oxidation reaction

WO_3	1 mg mL^{-1} in acetone + I_2	Carbon paper	Cathode	50	60	Stable photoanode for oxidation reaction
CeO_2	1 mg mL^{-1} in acetone + I_2	Carbon paper	Cathode	50	30	Functional oxide support materials electrode
Ta_3N_5	0.8 mg mL^{-1} in acetone + I_2	ITO and Carbon paper	Cathode	40	120	Visible-light photoanode for oxidation reactions
**Chalcogenides**						
MoS_2	1 mg mL^{-1} in acetone + citric acid	ITO and Carbon paper	Anode	60	45	Electron transport layer/Co-catalyst used in photoanode
WS_2	1 mg mL^{-1} in acetone + citric acid	ITO and Carbon paper	Anode	60	60	Electron transport layer/Co-catalyst used in photoanode
**MOF/COF materials**						
NU-1000 MOF	1 mg mL^{-1} in acetone + I_2	ITO	Cathode	10	90	Porous catalytic framework for photocatalysis
COF-366-Co	0.3 mg mL^{-1} in acetone	Carbon paper	Anode	30	180	Cathode for electrochemical CO_2 reduction
COF-366-Co/GNFs	0.083 mg mL^{-1} in acetone + I_2	Carbon paper	Cathode	60	60	Cathode for electrochemical CO_2 reduction
COF-366-Co/MWCNTs	0.25 mg mL^{-1} in acetone + I_2	Carbon paper	Cathode	60	300	Conductive COF/carbon composite for electrochemical CO_2 reduction

Unless otherwise stated, EPD was carried out in a two-electrode configuration at an electrode spacing of 2 cm under constant applied voltage. The deposition electrode indicates the electrode on which the film was collected under the optimised conditions. ITO = indium tin oxide; GNF = graphitic nanofibre; MWCNT = multi-walled carbon nanotube; MOF = metal–organic framework; COF = covalent organic framework.

Table S2. Quantitative tape-peel adhesion assessment of representative EPD-coated films.

**Material**	**Substrate**	**Initial film loading ($mg\ cm^{-2}$)**	**Film loading after tape test ($mg\ cm^{-2}$)**	**Mass retention (%)**
Ta_3N_5	ITO	0.33	0.05	15
Ta_3N_5	Carbon paper	0.32	0.28	87.5
g-C_3N_4	ITO	0.27	0.02	7.4
g-C_3N_4	Carbon paper	0.25	0.23	92
